# Structural features of somatic and germline retrotransposition events in humans

**DOI:** 10.1186/s13100-025-00357-w

**Published:** 2025-04-22

**Authors:** Päivi Nummi, Tatiana Cajuso, Tuukka Norri, Aurora Taira, Heli Kuisma, Niko Välimäki, Anna Lepistö, Laura Renkonen-Sinisalo, Selja Koskensalo, Toni T. Seppälä, Ari Ristimäki, Kyösti Tahkola, Anne Mattila, Jan Böhm, Jukka-Pekka Mecklin, Emma Siili, Annukka Pasanen, Oskari Heikinheimo, Ralf Bützow, Auli Karhu, Kathleen H. Burns, Kimmo Palin, Lauri A. Aaltonen

**Affiliations:** 1 Applied Tumor Genomics Research Program, Research Programs Unit, University of Helsinki, Helsinki, 00014 Finland; 2 Department of Medical and Clinical Genetics, Medicum, University of Helsinki, Helsinki, 00014 Finland; 3 Institute for Molecular Medicine Finland (FIMM), University of Helsinki, 00014 Helsinki, Finland; 4 Department of Pathology, Dana-Farber Cancer Institute, Harvard Medical School, Boston, MA 02115 USA; 5 Department of Computer Science, University of Helsinki, Helsinki, 00014 Finland; 6 Department of Gastrointestinal Surgery, Helsinki University Central Hospital, University of Helsinki, Helsinki, 00290 Finland; 7 Faculty of Medicine and Health Technology, University of Tampere and TAYS Cancer Centre, Tampere, 33100 Finland; 8 Department of Gastroenterology and Alimentary Tract Surgery, Tampere University Hospital, Tampere, 33520 Finland; 9 Abdominal Center, Helsinki University Hospital, Helsinki University, Helsinki, 00290 Finland; 10 iCAN Digital Precision Cancer Medicine Flagship, University of Helsinki, Helsinki, 00290 Finland; 11 Department of Pathology, HUS Diagnostic Center, Helsinki University Hospital and University of Helsinki, Helsinki, 00290 Finland; 12 Department of Surgery, Wellbeing Services County of Central Finland / Hospital Nova of Central Finland, Jyväskylä, 40620 Finland; 13 Department of Science, Well Being Services County of Central Finland, Jyväskylä, 40620 Finland; 14 Department of Health Sciences, Faculty of Sport and Health Sciences, University of Jyväskylä, Jyväskylä, 40014 Finland; 15 Department of Obstetrics and Gynecology, University of Helsinki and Helsinki University Hospital, Helsinki, 00290 Finland; 16 Department of Pathology, Mass General Brigham and Harvard Medical School, Boston, MA 02115 USA

**Keywords:** Retrotransposition, Transposable element, L1, Colorectal cancer, Uterine leiomyoma, Long read sequencing

## Abstract

**Background:**

Transposons are DNA sequences able to move or copy themselves to other genomic locations leading to insertional mutagenesis. Although transposon-derived sequences account for half of the human genome, most elements are no longer transposition competent. Moreover, transposons are normally repressed through epigenetic silencing in healthy adult tissues but become derepressed in several human cancers, with high activity detected in colorectal cancer. Their impact on non-malignant and malignant tissue as well as the differences between somatic and germline retrotransposition remain poorly understood. With new sequencing technologies, including long read sequencing, we can access intricacies of retrotransposition, such as insertion sequence details and nested repeats, that have been previously challenging to characterize.

**Results:**

In this study, we investigate somatic and germline retrotransposition by analyzing long read sequencing from 56 colorectal cancers and 112 uterine leiomyomas. We identified 1495 somatic insertions in colorectal samples, while striking lack of insertions was detected in uterine leiomyomas. Our findings highlight differences between somatic and germline events, such as transposon type distribution, insertion length, and target site preference. Leveraging long-read sequencing, we provide an in-depth analysis of the twin-priming phenomenon, detecting it across transposable element types that remain active in humans, including *Alus*. Additionally, we detect an abundance of germline transposons in repetitive DNA, along with a relationship between replication timing and insertion target site.

**Conclusions:**

Our study reveals a stark contrast in somatic transposon activity between colorectal cancers and uterine leiomyomas, and highlights differences between somatic and germline transposition. This suggests potentially different conditions in malignant and non-malignant tissues, as well as in germline and somatic tissues, which could be involved in the transposition process. Long-read sequencing provided important insights into transposon behavior, allowing detailed examination of structural features such as twin priming and nested elements.

**Supplementary Information:**

The online version contains supplementary material available at 10.1186/s13100-025-00357-w.

## Background

Transposable elements (TEs) are DNA sequences with the ability to move or copy into other locations of the genome [1]. Of the TEs in the human genome, the only currently known active elements are retrotransposons. They make new copies of themselves via an RNA intermediate [2] and can be further classified into autonomous and non-autonomous elements [3]. Autonomous elements encode for all proteins necessary for mobilization, whereas non-autonomous elements utilize the proteins encoded by autonomous TEs. The only actively retrotransposing autonomous retrotransposons in the human genome are Long interspersed nuclear element-1s (L1s), whereas the only active non-autonomous elements are short interspersed nuclear elements (SINEs), which can be further classified based on their sequence similarities into *Alus* and SINE-Variable number tandem repeat (VNTR)-*Alu* (SVA)-elements. As non-autonomous elements, SINEs use the L1 protein ORF2p to retrotranspose [4, 5, 6]. In addition to SINEs, L1 proteins can also retrotranspose other mRNAs originating from protein coding genes resulting in processed pseudogenes (PP) [7]. Along with L1s, endogenous retroviruses (ERV) are autonomous retrotransposons, however there is no record of current ERV retrotransposition activity in humans [8].

The canonical L1 retrotransposition mechanism is target-primed reverse transcription (TPRT) [9]. TPRT starts with transcription of the L1 element in the nucleus of the cell. After translation of the two open reading frames (ORF1p and ORF2p), the L1 RNA, ORF1p, and ORF2p form a ribonucleoprotein which re-enters the nucleus for integration of the L1 RNA. In the nucleus, the endonuclease (EN) domain of ORF2p, nicks the genomic DNA at a consensus target sequence 5′-TTTT/A(A)-3′ [10] providing a primer for the reverse transcriptase (RT) domain of ORF2p. The polyA-tail of the L1 RNA hybridizes with the DNA overhang containing the tract of Ts and is used as a template for the first strand DNA synthesis of the new TE insertion [11]. In most insertions, the complementary DNA strand is cleaved downstream of the EN cut site, causing the sequence between the nicks to flank the L1 insertion on both sides, leading to target site duplications (TSD) [10, 12]. TPRT continues with the synthesis of the second DNA strand by ORF2p and poorly understood host-catalyzed processes may also contribute [13]. Retrotransposon insertions resulting from TPRT display common characteristics known as hallmarks of retrotransposition. Most common hallmarks include insertion breakpoints at EN consensus cut sites, TSDs flanking the insertions, polyA-tails at the 3′ end of the insertions, incomplete insertions known as 5′ truncations, and, less frequently, internal 5′ inversion of the inserted sequence. The proposed mechanism for the formation of a 5′ inverted sequence is twin priming, a variation of TPRT, where reverse transcription of the L1 RNA is primed by both DNA strands resulting in an insertion with a 5′ inversion [14].

There are approximately 500,000 mostly truncated L1 elements in the human genome [15], but only ~ 100 per genome are estimated to be potentially active [16, 17]. In healthy adult tissues, retrotransposons are repressed by epigenetic silencing [18], with low levels of activity in some tissues such as brain [19, 20, 21] and recently discovered colon [22]. In addition, retrotransposons become very active in many human cancers, especially in cancers of epithelial origin, such as head and neck, esophageal and colorectal cancers (CRCs) [23, 24, 25, 26, 27, 28]. However, more studies are needed to understand the impact of somatic retrotransposons across the manifold of tumors. For example, somatic retrotransposon activity in benign tumors and tumors of mesenchymal origin has remained poorly understood. In cancers, most somatic insertions are intronic or intergenic with an unclear effect on gene expression [23], however approximately 1% of colorectal cancers are initiated by somatic retrotransposon insertions disrupting the *APC* gene [28, 29, 30]. Furthermore, the number of retrotransposon insertions correlates with poor survival in colorectal cancer [28].

Beyond somatic expression, TEs occasionally exhibit germline activity, although insertions occurring in germline lineages are hard to distinguish from somatic insertions occurring prior to germline partitioning in early embryogenesis. The estimated rate of novel *Alu* insertions is 1 in 20–40 live births [31, 32], while the insertion rate for L1 elements is smaller, being 1 in 60–270 births [32, 33, 34]. This aligns with the observation that *Alus* are the most prevalent TEs in the human genome in terms of the number of insertions [15]. However, paradoxically, L1s account for the majority of somatic insertions observed in cancers [23, 26, 35].

In this study, we examined CRCs, malignancies of epithelial origin, uterine leiomyomas (UL), monoclonal but benign tumors of mesenchymal origin, and adjacent normal tissues, to detect somatic TE insertions and germline TE polymorphisms. We analyzed 56 CRC and 112 UL long read sequencing samples, detecting 1495 somatic TE insertions from CRCs, but no evidence of somatic activity in ULs, despite demonstrable sensitivity. Altogether, we identified 7265 distinct germline TE polymorphisms in both CRC and UL patients. Additionally, we detected one somatic processed pseudogene insertion, and 22 germline processed pseudogene polymorphisms. We show that insertions tend to be located within their own types of reference genome TEs and that these are associated with replication timing. Moreover, we characterized the detailed sequence features of insertions with twin priming and identified differences between somatic and germline events, suggesting distinct genomic mechanisms influencing them.

## Results

### Characterization of retrotransposon insertions in long read whole genome sequencing

**Fig. 1:**
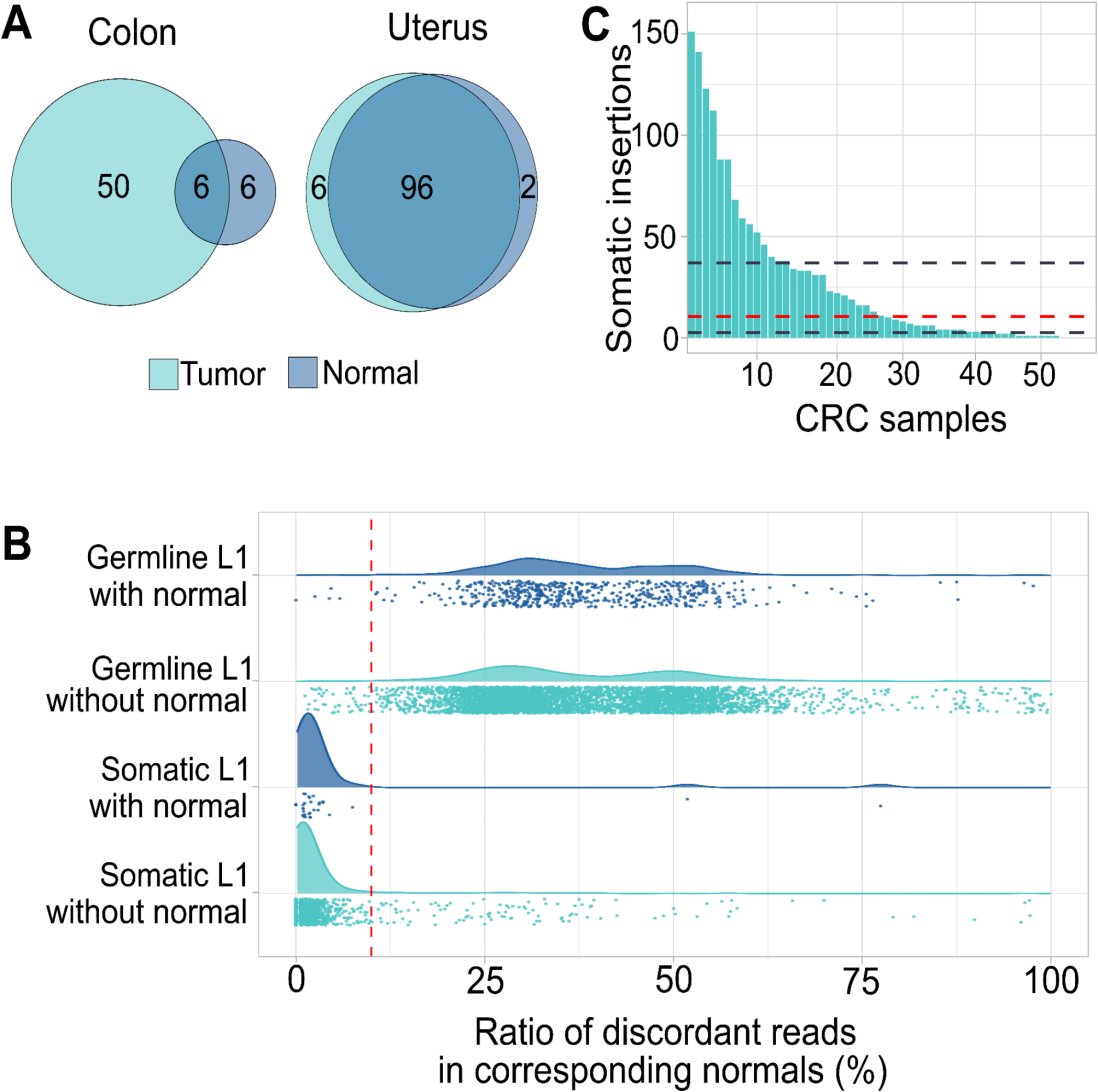
Overview of samples and distribution of insertions. (**A**) Venn diagram of individuals with both tumor and corresponding normal samples, with only normal samples and with only tumor samples in CRC and UL data set. (**B**) Ratio of discordant Illumina read pairs in normal colon samples around nanopore called L1 elements. Strip plot and kernel density estimates of ratio of discordant reads to all reads within 100 bp of insertion site. The insertions are divided into groups based on the somatic filtering and the presence of corresponding normal samples sequenced with Nanopore. The red dashed line marks the threshold of 10% discordant read rate. (**C**) Distribution of somatic L1 insertion counts in CRC samples. Grey dashed lines are the 1st and 3rd quartile, and the red line is the median of insertions per sample

In this study we set out to detect and characterize somatic and germline TEs from CRC and UL samples utilizing nanopore sequencing. Our whole genome sequenced set of 280 samples from 168 different individuals consisted of 112 ULs, 98 normal myometrium, 56 CRC and 12 normal colon samples (Fig. 1a). Six individuals had 2 UL and two had 3 UL sequenced. Most UL samples had a corresponding normal sample, with 6/112 tumors having no normal sequenced. In contrast 6/56 CRCs had a nanopore sequenced corresponding normal. However, utilizing a pool of normals in somatic filtering resulted in a high confidence somatic call set (Fig. 1b) as demonstrated by validation with short read sequenced corresponding normal DNAs.

We used a custom pipeline (Methods TE detection pipeline) and detected 7265 germline TE polymorphisms from CRC and UL samples and 1495 somatic L1 TE insertions from CRC samples (Additional Table S1). In addition, we detected 23 processed pseudogenes: 22 germline polymorphisms and one somatic insertion in CRC (Additional Table S1).

We evaluated the performance of our TE detection pipeline by analyzing the Genome in a Bottle sample HG002 [36] and four CRC samples using xTea [37] (Additional methods). Both pipelines detected similar TE events, with our method identifying 89% of benchmarked HG002 insertions and xTea detecting 96%. Both pipelines respectively detected 15% and 32% insertions that were not benchmarked (Additional Table S2). In the CRC dataset, 57% of the TE calls were detected by both methods, with xTea detecting 74% (on average 616/830 per sample) of the remaining calls. Based on manual evaluation of the presence of hallmarks in the unique calls we estimated that 46% (23/50) of xTea calls and 70% (35/50) of our calls were true TEs (Additional table S3). Based on these numbers, both methods mainly detected the same events, with xTea being more sensitive and our calls more specific (Additional methods).

Next, we focused on evaluating the performance of our TE detection pipeline with somatic calls. For CRC samples, most of which lacked corresponding normal tissues (Fig. 1a), we compared the number of somatic insertions to the expected number of singleton germline events (Methods TE type based somatic filtering). Only L1 insertions exceeded the expected number of singleton germline polymorphisms, suggesting they are mainly true somatic events. This resulted in exclusion of all non-L1 insertions from the somatic call set (Methods Additional filtering), consistent with prior studies showing that somatic insertions are primarily L1 insertions [23, 25, 26, 28]. To assess the true somatic rate of the L1 calls, we analyzed the presence of discordant reads in Illumina short-read data from corresponding normal samples available for 54 of the 56 CRCs included in this study. 93% of somatic calls exhibited fewer than 10% discordant reads in the normal sample, in contrast to just 1% of germline calls (Fig. 1b, Methods: Manual curation, Additional Table S4). This suggests that approximately 93% of the somatic calls are likely true somatic, and that the reliability of our somatic calls is not significantly affected by the presence of a corresponding normal sample. In addition, a subsequent blinded manual evaluation of 50 random insertions (Methods Manual curation, Additional Table S5) gave a similar true somatic rate of 96%. Further, to utilize the power of long reads, we performed graph analysis where we aligned the nanopore reads to sequence graphs of insertion and reference alleles (Methods Graph analysis and detection of supporting reads). We compared the number of reads aligning to the insertion graph to the reference graph for all L1 insertions in six CRC patients with both tumor and normal nanopore sequenced. All the somatic L1 insertions showed reads aligning to both the insertion and reference allele, but in the normal the reads supported only the reference allele (Additional Figure S1). This provides further evidence of the somatic nature of our calls.

To compare our somatic calls with short read sequencing, we compared L1 calls from 50 CRC samples using nanopore sequencing and our pipeline and Illumina sequencing and xTea. The different datasets contained 36% of the same TE calls. As samples sequenced with short-read technology had higher coverage and more molecules sampled than those sequenced with Nanopore, we initially expected to identify more insertions in the short-read data. Other factors may also contribute to differences in insertion detection, including methodological variations and the use of different filtering criteria specific to each sequencing approach. Notably, 26% of the somatic insertions identified using long-read sequencing were not detected in the short-read data, likely due to their location within repetitive regions of the reference genome (Additional Methods). This indicates that long read sequencing has access to TE insertions previously left undetected by short read sequencing, as has been previously shown [38].

To validate our findings, we performed PCR and Sanger sequencing for 13 somatic L1 insertions from 12 samples (Methods PCR and Sanger validation, Additional Table S6). The primer pairs consisted of one pair spanning the breakpoint or covering the inserted sequence and the other outside the insertion, leading to products specific to the insertion. All the PCR reactions resulted in a band of expected size in the tumor (Additional Figure S2a and S2b) and Sanger sequencing of the PCR products showed that the sequences of the products were similar (90–100% similarity) to sequences identified by the pipeline (Additional Table S6, Additional File 3). In 10 of the insertions, there were no bands in the corresponding normal when running the PCR in the same conditions (Additional Figure S2a). Three insertions, however, produced a faint band in the normal (Additional Figure S2b). As the normal colon samples lacked reads supporting them in Illumina (~ 40 X) or Nanopore (~ 20 X) WGS when visually validating them (e.g. Additional Figures S3, S4 and S5), and the PCR products appeared visibly fainter in the normal samples (Additional Figure S2b), we suspected somatic mosaicism as the underlying cause. A pathologist review of the histology of the available normal sample decreed it not containing tumor; however, a possibility of contamination cannot be ruled out (Additional Methods: Additional Notes on PCR). An alternative explanation is an unspecific PCR, however, the Sanger sequenced normal and tumor sequences were identical to each other containing both insertion and > 50 bp of specific reference sequence as expected (Additional Table S7). Overall, PCR validation supports that the detected insertions are true somatic events and that the sequence prediction was successful. Furthermore, it suggests possible somatic mosaicism in the normal colon.

We detected a total of 1495 somatic L1 insertions in CRC samples (Additional Table S8). Somatic insertion count varied greatly between CRC patients, with a mean of 26.7 and a median of 9.5 somatic insertions per sample [Quartiles 2.75, 34.75, max 151] (Fig. 1c). Only five samples had no somatic insertions detected in them. Germline transposable elements (TEs) were compared to the human TE database HMEID [39], using a window of 300 bp and demanding the TE type to match. This revealed that 58% (4191/7265) of germline TEs that we detected are already catalogued in the database. The remaining TEs, which are not present in HMEID, tend to be rare: 33% (1015/3074) were detected in three or fewer individuals. In contrast, 20% (850/4191) of the previously reported TEs show similar rarity. The most common of not previously reported TEs—those found in at least 90% of individuals (150/166)—are predominantly located within annotated repeat regions in the reference genome, with 78% (62/79) overlapping known repeats.

### Absence of somatic TE insertions in uterine leiomyomas

Uterine leiomyomas (ULs) are benign clonal smooth muscle tumors that affect 70% of premenopausal women [40]. In up to 25% of women ULs cause significant morbidity, including pain, pressure and abnormal menstrual bleeding, resulting in substantial economic and quality of life burden [41]. ULs are characterized by specific genetic alterations, such as frequent *MED12* mutations, *FH* inactivation, *HMGA2* rearrangements, and chromosomal abnormalities [42]. Although the genetic landscape of ULs has been extensively studied [43, 44, 45], the role of TE insertions in their pathogenesis remains unknown. To investigate the presence of somatic TE insertions in ULs, we analyzed short-read and long-read whole genome sequencing data using three different detection methods. First, we applied our TE detection pipeline to Nanopore long-read whole genome sequencing data from 112 uterine leiomyomas and 98 normal myometrial tissues (Fig. 1a). As somatic TE insertions have not been reported in UL, we included *Alu* elements and insertions without hallmarks of retrotransposition to increase sensitivity. We called 183 somatic TE insertions (Additional table S9), however, upon closer inspection (Methods Manual curation), the called insertions were deemed as either false positives or germline events.

To further explore the possibility of TE insertions in ULs, we employed an alternative detection method, xTea, on the same Nanopore data. xTea successfully processed 41 samples, identifying 311 calls (Additional Figure S6). Most of them appeared germline, as 232/311 (75%) had a notable ratio (≥ 10%) of discordant reads in the corresponding normal, and of the remaining calls, only 5/79 (6%) had both a polyA-tail and a TSD of 5–25 bp detected (Additional table S9). Only 8/311 calls overlapped with calls detected by our pipeline and in manual curation these insertions were classified as germline based on the presence of supporting reads in the normal sample (Methods Manual curation).

To further corroborate these findings, we analyzed xTea calls from Illumina short-read sequencing data from 8 UL samples. xTea detected 120 somatic L1 and 1315 somatic *Alu* insertions (Additional Figure S6); however, retrotransposition hallmarks (both polyA-tail and TSD of 5–25 bp) were identified in only 55 of the combined 1435 calls, indicating that most were unlikely to be genuine retrotransposition events (Additional table S9). Moreover, none of the 1435 TE calls from short read data overlapped with any of the 17 TE calls detected from the same samples from Nanopore data (Additional Figure S6). In contrast, using xTea with Illumina data and our method on CRC samples, we detected on average 21 shared somatic TEs per sample with both methods (Additional methods), demonstrating that these pipelines can identify the same true somatic insertions with different sequencing types and coverages. Thus, the absence of insertions detected by long and short read sequencing in ULs suggests that the detected insertions in these tumors are not genuine somatic events. Additionally, 3392/8940 (38%) calls exhibited a high ratio (≥ 10%) of discordant reads in matched normal samples, supporting their classification as germline rather than somatic insertions (Additional table S9, Methods Supporting reads in normal samples). Furthermore, somatic *Alu* insertions are rarely observed in cancer genomes, underscoring how unlikely their presence is in ULs [23, 25, 26].

Our research found no evidence of somatic TE insertions in uterine leiomyomas. While our analysis initially identified potential somatic insertions using three detection approaches (Our TraDetIONS pipeline and xTea on long reads and xTea on short reads), upon closer inspection most of these (38–75%) were actually germline variants present in normal tissue (Additional table S9). The remaining candidates lacked typical hallmarks of retrotransposition, showed unusually high rates of *Alu* insertions, and had minimal overlap (< 3%, Additional Figure S6) between detection methods. We conclude that the rate of somatic TE insertions in uterine leiomyomas is below our limit of detection.

### Somatic L1s exhibit shorter lengths and higher hallmark frequencies compared to germline L1s

To explore the differences between TE types and between germline and somatic retrotransposition we compared the frequency of retrotransposition hallmarks in insertions arising from L1s, *Alus*, SVAs and PPs (Table 1).

**Table 1:** Comparison of L1 Retrotransposition hallmarks across TE types encoded by the L1 ORF2p. PPs consist of both germline (22) and somatic (1) PPs. Hallmarks of Retrotransposition include: insertion length (median length), the percentage of insertions with a detected polyA-tail, polyA-tail median length (bp), target site duplication or deletion (defined as 5–25 bp overlap or distance between insert flanking sequences on the reference), EN cut site (TTTT/A, TTTT/G, TTTC/A) and twin priming events

	Total (*n*)	Median length (bp)	Poly-A tail (%)	Poly-A tail median length (bp)	TS duplication (%)	TS deletion (%)	EN cut site (%)	Twin priming rate (%)
somatic L1	1495	394	91.6	39	65.4	8.4	70.1	21.9
germline L1	897	1240	90.7	34	69.5	7.4	63.4	18.4
germline *Alu*	5936	320	95.1	39	83.8	2.3	62.7	0.02
germline SVA	322	1154	91.3	36	71.7	7.0	69.3	0
PPs	23	1373	95.7	27	69.6	4.3	69.6	13.0

**Fig. 2:**
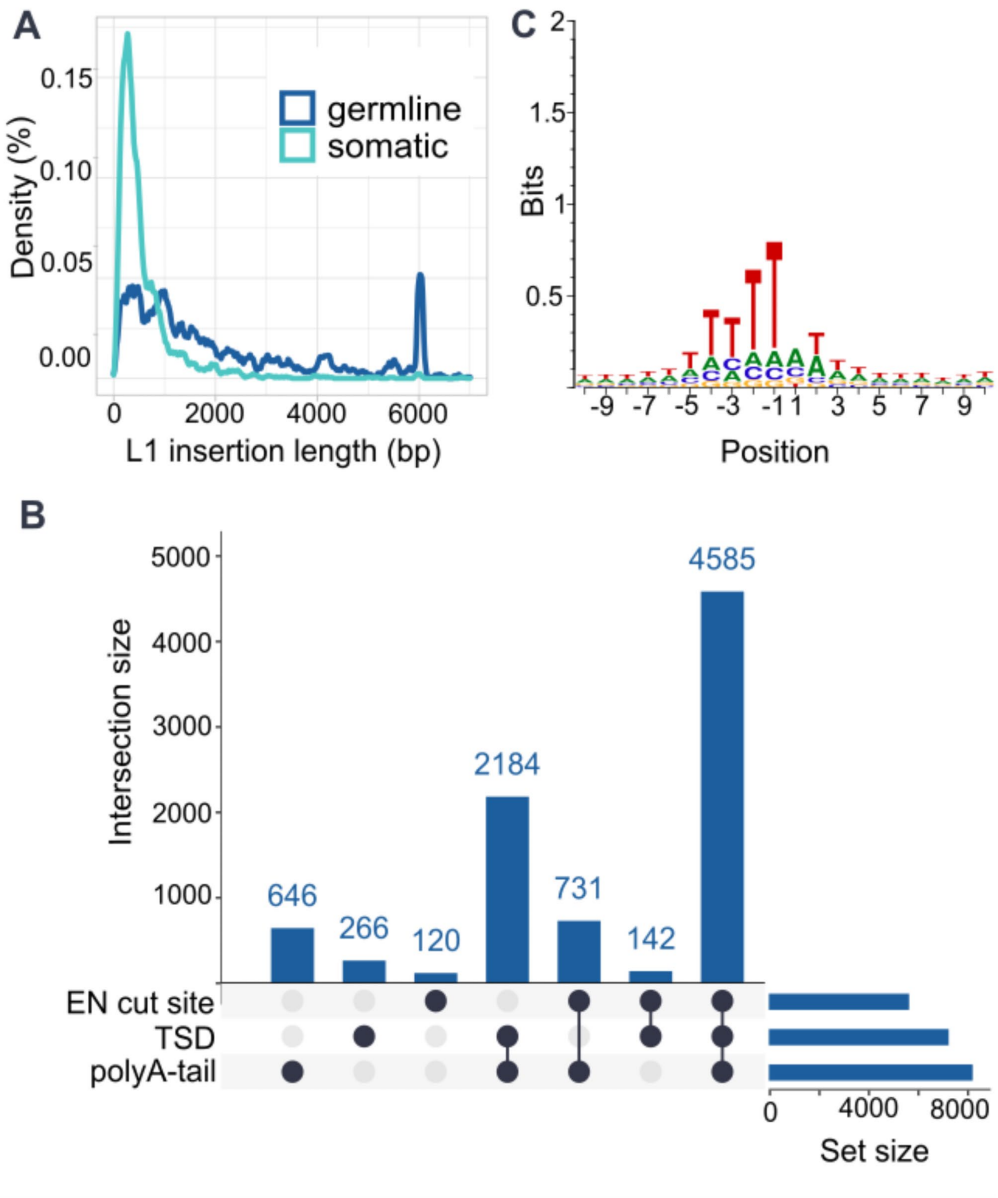
Overview of retrotransposition hallmarks (**A**) Lengths of L1 insertions as rectangular kernel density estimate with bandwidth of 40 bp. (**B**) UpSet plot of presence of hallmarks of retrotransposition in all TPRT-related insertions (**C**) Base composition within 10 bp of the first strand nick in all TPRT-related insertions. The nick occurs between positions − 1 and 1

Most germline events were identified as *Alu* (5936/7265) in contrast to somatic insertions, where we could confidently only detect L1s (Additional Table S1, Methods TE type based somatic filtering). This is concordant with previous studies where 94–99% of somatic insertions were from L1 elements [23, 25, 26, 28]. As expected, insertion length varied between different TE types, and somatic L1 insertions were significantly shorter (median 394 bp) than germline L1 polymorphisms (median 1240 bp) (*p* < 2.2✕10^− 16^, two-sided t-test, Table 1). In addition to this difference, 8.7% (78/897) of germline L1 polymorphisms were full length (> 5990 bp), whereas only 0.1% (2/1495) of somatic insertions were full length (*p* < 0.00001 Chi-square test, Fig. 2a). These results are also concordant with previous studies where somatic L1 insertions are significantly shorter than germline events [22, 23, 46].

Most of the insertions contained a poly-A tail, a target site duplication or deletion, and an endonuclease cut site (Fig. 2b). The most frequently detected hallmark was the polyA-tail which was detected in 94% of the TEs, being the highest in germline polymorphic *Alus* and processed pseudogenes (Table 1). The median length for polyA-tail was 38 bp with no significant differences between TE types (Additional table S4). We identified target site duplications in 79% (6826/8673) of the calls and identified a target site deletion in only 4% (350/8673).

L1 elements and PPs have been reported to experience 5′ inversions, a phenomenon proposed to result from twin priming [14, 47, 48]. We detected it from both germline and somatic L1 elements with a slightly higher rate in somatic (22%, 328/1495) than in germline L1s (18%, 165/897) (*p* = 0.038, Chi Square test) (Table 1). Additionally, we detected 5′ inversions in PPs with a rate of 13% (3/23). In addition to L1 elements and PPs, both *Alu* and SVAs retrotranspose utilizing the L1ORF2p, suggesting that they could go through the same phenomenon. In the PCAWG dataset they report a low rate of 5′ inversions in somatic *Alus* (1/130, 0.8%) and SVAs (2/23, 9%) [26], however germline assessment of twin priming in these elements remains elusive. After systematic detection and curated evaluation of inversions in germline *Alu* and SVA, we confidently detect one *Alu* element with a 5′ inversion (Additional File 2), however no 5′ inversions were found in SVA elements. Thus, the observed twin priming rates for germline *Alus* and SVAs are 0.02% and 0%, respectively. This low frequency aligns with the limited reports of inversions in *Alu* and SVA elements [26]. However, it remains unclear why the rates of twin priming are as different across TE types. One possible explanation is the element length: *Alu* elements are shorter than L1 elements, reducing the likelihood of twin priming during transcription. However, somatic L1 elements are significantly shorter than germline L1 elements and PPs, yet they exhibit the highest rate of twin priming (Table 1). This suggests that whilst the element length may play a role, other factors are likely involved in determining the occurrence of twin priming.

The endonuclease cut site was detected in 64% (5578/8673) of the studied insertions (Table 1). The highest rate of endonuclease cut sites was found in somatic L1s (1048/1495 = 70%) which is significantly higher (*p* < 0.000744, Chi Square test) than germline L1s (569/897 = 63%) (Table 1). To model the EN cut site motif at the insertion breakpoints in finer detail, we examined the base composition for each base within 10 bp both up and downstream of the first strand nick identifying a position weight matrix corresponding to the canonical EN motif (Fig. 2c).

To demonstrate the recent advancements in Nanopore sequencing, we detected TEs in 13 UL samples sequenced using the R10 nanopore technology and the T2T-CHM13v2.0 reference genome [49]. From this dataset, we identified 3478 TEs and 19 PPs that all were likely germline (Additional Table S10). The use of more recent and accurate sequencing chemistry resulted in a slight improvement across all quality metrics, including higher target site duplication rates, increased EN cut site identification, and improved poly-A tail detection rate (Additional Table S11).

### Comparison of insertions with 5′ inversions in somatic and germline L1 insertions

**Fig. 3:**
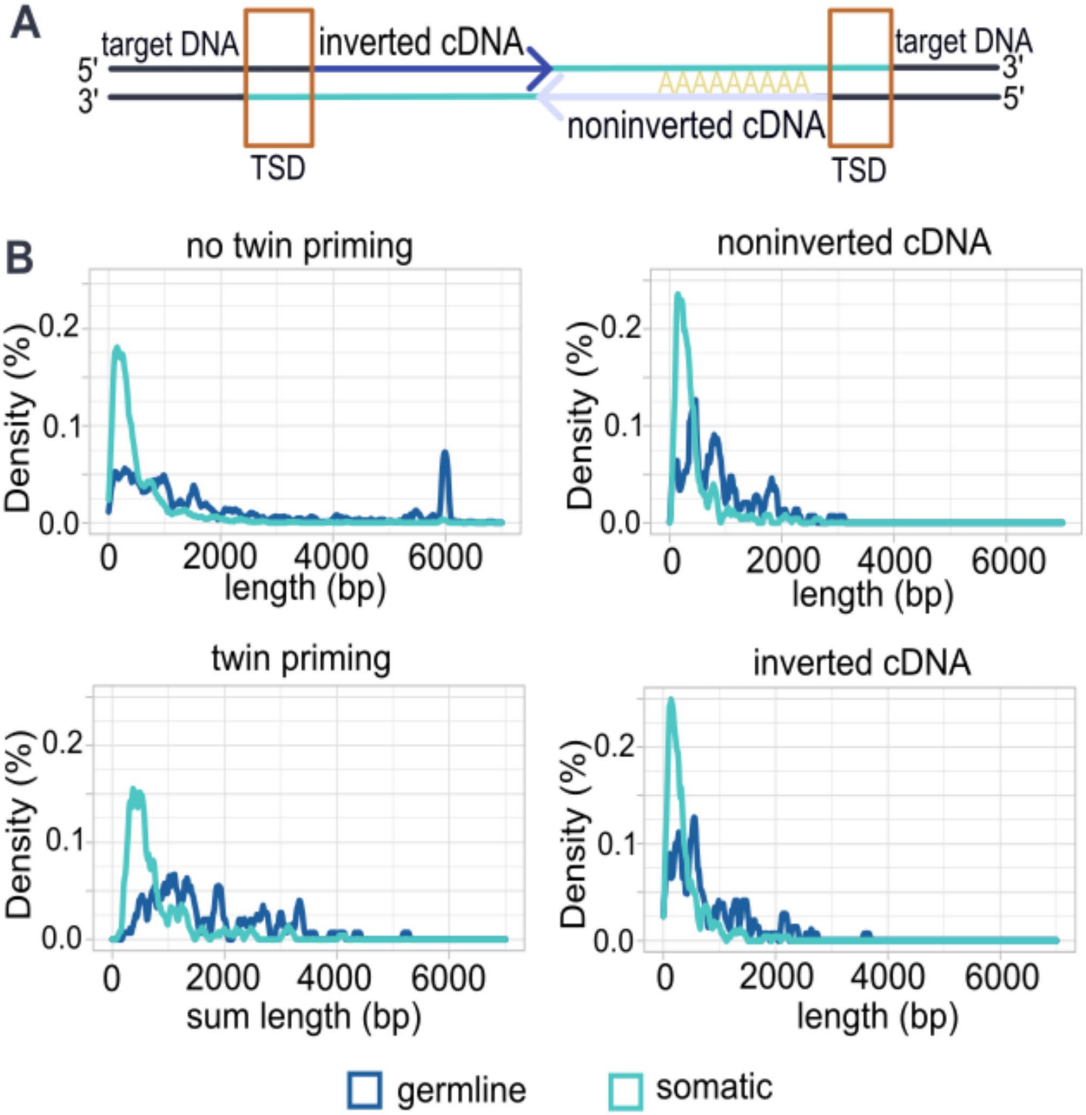
Characteristics of 5′ inverted insertions (**A**) Schematic of an insertion with 5′ inversion consisting of noninverted cDNA (polyA-primed) and inverted cDNA (internally primed) with opposite orientations. (**B**) Length of L1 insertions with and without 5′ inversion and cDNAs forming the 5′ inverted insertion in somatic and germline events

We detected a 5′ inversion in 21.9% of somatic L1 insertions and in 18.4% of germline L1 polymorphisms. A proposed mechanism for the 5′ inversions is twin priming [14], however the mechanism and detailed characteristics of these insertions have remained poorly studied. With twin priming, the insertion consists of two separately polymerized cDNAs with opposite orientations: the usual noninverted cDNA that is primed from the polyA-tail of the L1 mRNA, and the inverted cDNA that is primed from an internal sequence of the L1 (Fig. 3a).

To elucidate the characteristics of these insertions, we first compared the lengths of the individual cDNAs and their sum, with the length of insertions without 5′ inversions (Additional Table S12). In somatic insertions with 5′ inversion, both the noninverted and inverted cDNAs had similar lengths, with a median of 253 bp and 292 bp respectively. However, the sum length of cDNAs in insertions with 5′ inversion (median 541 bp) tends to be longer than the cDNA of insertions without 5′ inversion (median length 308 bp) (Fig. 3b).

In germline polymorphisms with 5′ inversion, the cDNA lengths were generally longer than in somatic insertions, and with more variability. Inverted cDNAs were longer than noninverted insertions in germline, with a median of 751 bp and 571 bp respectively. In contrast to somatic insertions, germline polymorphisms with and without 5′ inversion were of similar total length (median length of 1363 bp and 1330 bp respectively) (Fig. 3b).

**Fig. 4:**
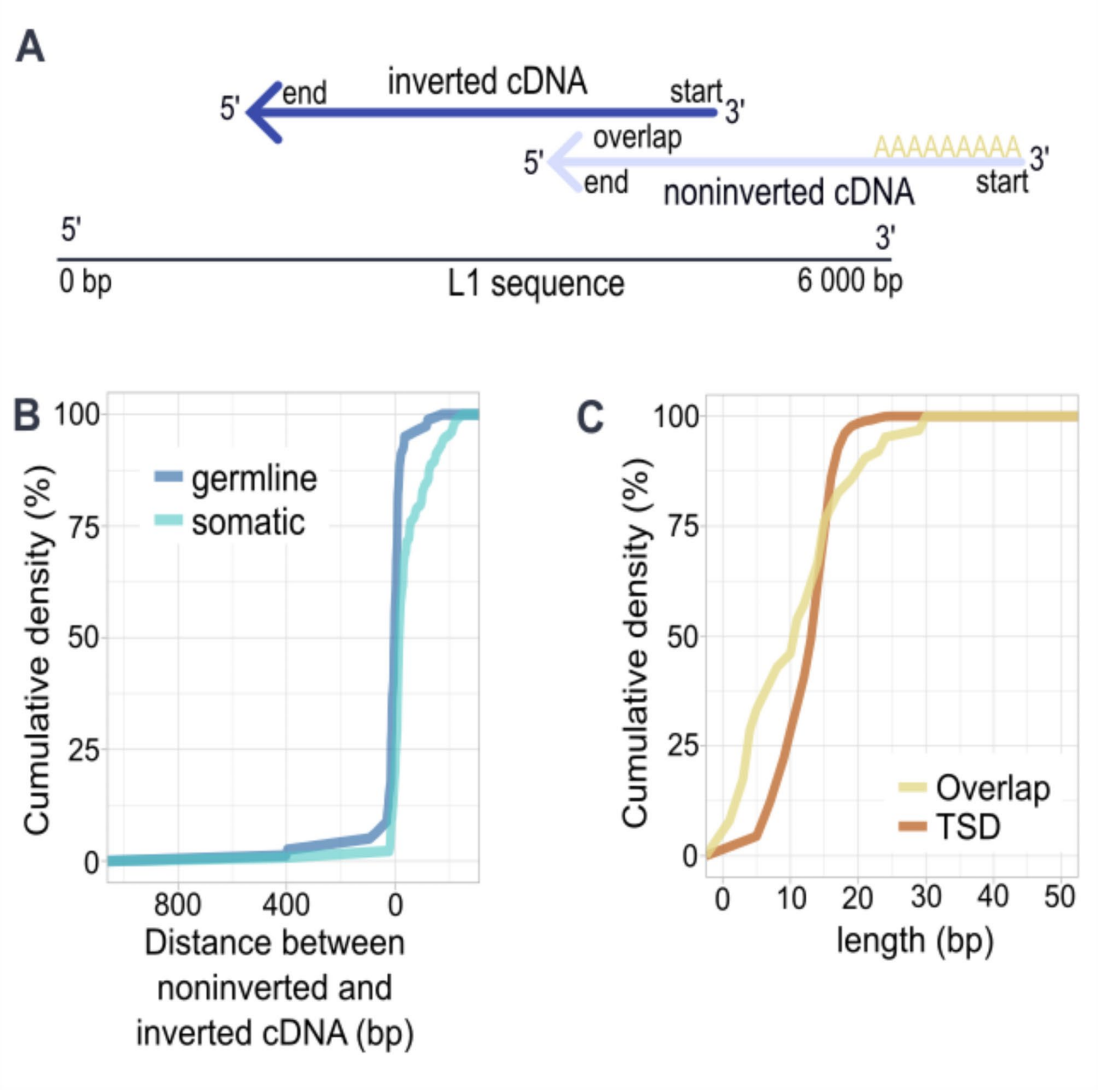
Distance between cDNA sequences in 5′ inverted insertions (**A**) Schematic of both the noninverted cDNA and inverted cDNA mapped to the L1 reference sequence. If the end of noninverted cDNA and start of inverted cDNA contain the same sequence an overlap occurs, as illustrated here. (**B**) Cumulative density function of the distance between noninverted cDNA end and inverted cDNA start. Positive distance represents a gap between the cDNAs, negative distance an overlap. (**C**) Cumulative density function of length of overlap between noninverted cDNA and inverted cDNA and target site duplications in L1 insertions

To further understand where the breakpoints of the 5′ inversion occurred, we aligned both the noninverted and inverted cDNA to the L1 reference sequence [50] (Fig. 4a) for 287 somatic and germline L1 calls containing 5´ inversions. We found that 46% (132/287) of the insertions had the end of noninverted cDNA and start of inverted cDNA within 20 bp of each other (Fig. 4b). In 41% (54/132) of them, the cDNAs overlapped, where both the end of noninverted cDNA and the start of inverted cDNA share the same sequence (Fig. 4a). Interestingly, we observed that an overlap occurred significantly more often in germline (42%, 44/106) than in somatic insertions (15%, 28/181) (*p* = 0.00001, Chi-square test). The length distribution of the cDNA overlap (median 12 bp, quartiles [4 bp, 17 bp]) is highly similar to the observed length of target site duplications (median 13 bp, quartiles [10 bp, 15 bp]) (Fig. 4c) which could suggest shared mechanisms. To explore whether the L1 EN could be implicated in producing both the TSD and the overlapping twin priming cDNA, we searched for the canonical EN cut site motif at the 5′ end of noninverted cDNA inside insertions with and without 5′ inversions. However, with the applied method we did not find additional EN motifs or recurrent patterns apart from the expected EN cut site at target priming site (Additional Figure S7).

We next studied whether the inverted cDNAs tend to be primed at specific locations within the L1 element (Fig. 4a). We analyzed three breakpoints involved in twin priming; the noninverted cDNA end, the inverted cDNA start and the inverted cDNA end. We observed that the breakpoints were distributed across the 3′ end of the L1 reference sequence and showed a pattern of peaks, indicating that certain regions were more prone to breakpoints (Additional Figure S8). The breakpoints in somatic insertions were more concentrated compared to germline, which could arise from somatic L1 elements arising from narrower selection of source L1 elements, creating a more homogeneous insertion pool. Although the L1 sequence does not vary significantly between the active L1 elements, epigenetic or downstream sequence transducing could have a role in the formation of the inversions [51].

### Somatic processed pseudogene insertions are rare events in colorectal cancers

To determine the somatic and germline frequency of processed pseudogene (PP) insertions that are not present in the reference genome, we extracted insertions containing cDNA sequences and hallmarks of retrotransposition (Methods TE detection pipeline). We detected a total of 23 polymorphic processed pseudogenes from which 22 were detected in the germline. We were able to detect only one somatic PP insertion, encountered in a tumor with high somatic L1 insertion count (143) (Additional Table S13).

The rate of somatic PPs insertions in our tumors (1.8%, 1/56) was similar to previous reports (2.6% 17/660) [47] suggesting that somatic retrotransposition of a non-transposon mRNA is a rare event. Half (11/22) of the germline PP polymorphisms have been reported in other datasets [52, 53, 54] (Additional Table S13). As expected, the previously reported PPs in our dataset were detected in more individuals (median in 13 out of 168 individuals) than PPs only detected in our study (median 1/168 individuals). This suggests that the previously known PP insertions are older and thus more common in humans.

### Germline TEs are enriched in repetitive DNA

To further understand insertion site differences between somatic and germline TEs, we compared the frequency of somatic insertions in genes, cancer genes, and annotated repeat sequences to the frequency of germline polymorphisms in the same target regions. While this comparison provides insight into potential differences in insertion site preferences, it is important to note that only germline insertions are subject to evolutionary selection pressures, which may influence their observed distribution.

We identified a similar frequency of TEs in genes (including exons, introns and untranslated regions) in both the germline (58.7%, 4281/7287) and soma (54.4%, 814/1496) (Additional Table S14) (GENCODE 39) [55]. In addition, we identified 124 cancer genes [56] with at least one germline and 23 cancer genes with at least one somatic event (Additional Table S14). Both somatic (1.0%, 15/1496) and germline (0.8%, 61/7287) TEs only rarely hit common fragile sites [57].

**Fig. 5:**
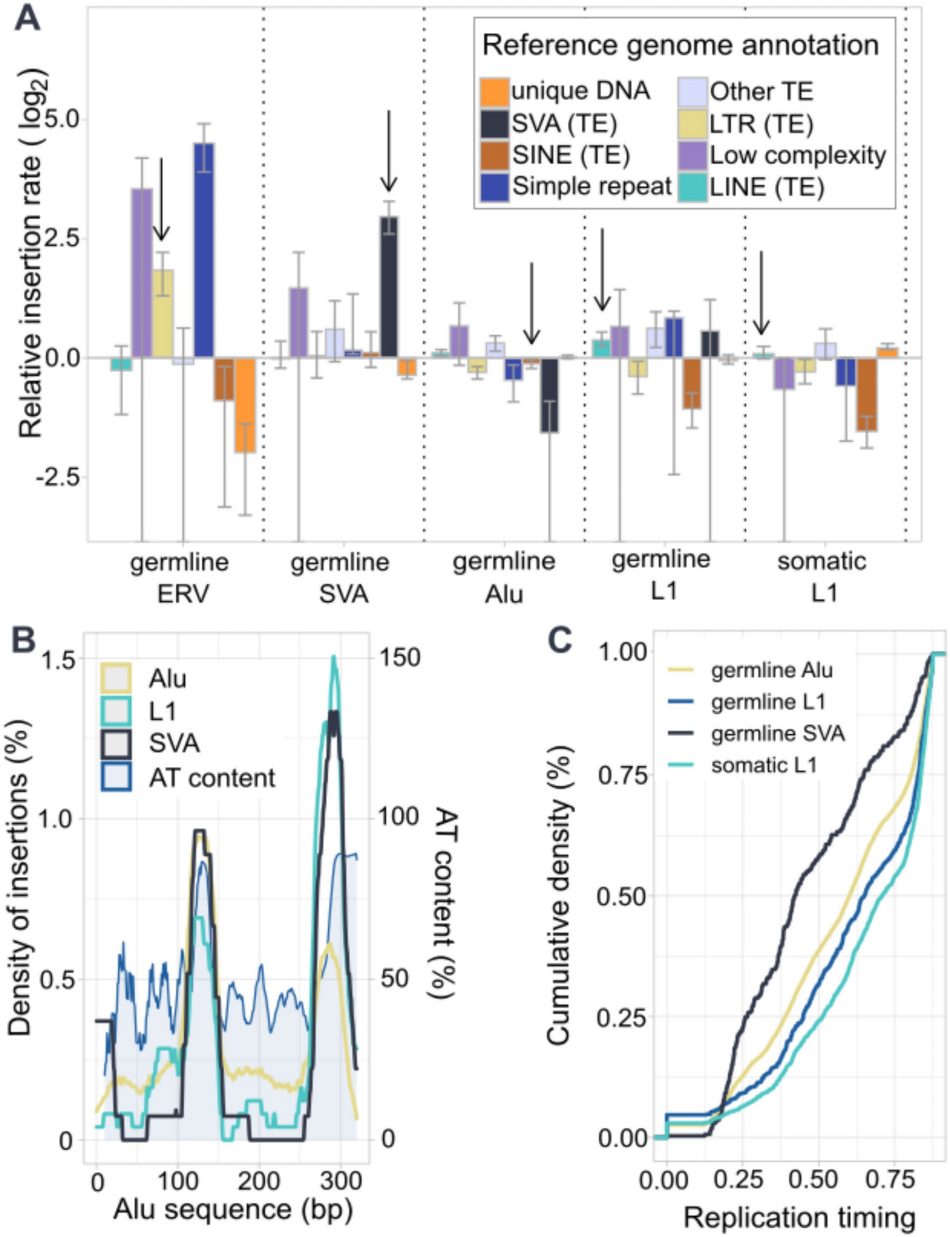
Insertion target sequence annotation (**A**) TE insertions prefer different types of reference with varying rates between TE type and soma/germline context. Arrows depict target annotations of the insertion′s own type. (**B**) Insertion loci inside reference genome *Alu* elements. The insertions are represented with a rectangular kernel density estimate with bandwidth 10 bp. AT content expresses the rate of A/T-bases in the positions inside reference *Alus* (smoothed with rolling mean k = 10). (**C**) Cumulative density function of replication timing of targets divided by insertion types

Next, benefiting from the mappability of long reads, we evaluated the potential target preference of somatic insertions and germline polymorphisms in different repeat areas in contrast to unique DNA. We compared the ratio of insertions from different TEs hitting different genomic areas and accounted for how prevalent these target areas are in the reference genome (Additional Table S15). We divided the reference genome into 8 annotation classes: LINE, LTR, SVA, SINE, Simple repeat, Low complexity, Other TE- including repeats outside previous classifications, and unique DNA- defined as the sequence outside the repeat annotations [58]. We found that all germline polymorphism types showed preference for Low complexity areas. Other germline polymorphisms than *Alus* show preference for simple repeats and TEs of their own repeat type, with the highest preference in SVA insertions within reference SVAs (Fig. 5a). Somatic L1 insertions preferred their own TE type, but also unique sequence (Fig. 5a).

To further explore TE insertion self-preference, we scrutinized whether particular regions within the repeat were targeted (Fig. 5b, Additional Figure S9). We analyzed only *Alus* and L1s since they were the most frequent targets, and from each of them we only selected subfamilies of similar lengths. To investigate the connection of the TE sequence content and insertion frequency we counted the AT content within the repeat sequences and the number of times a given base of the TE is present in the reference genome. Unlike L1 elements, which are often truncated at the 5´end and therefore overrepresented at the 3´ ends in the reference genome (Additional Figure S9b), *Alu* elements are typically present in full, resulting in an even representation of their entire sequence. When comparing the different *Alu* elements in the reference, there is a clear preference to insert in two areas of the sequence (positions 100–155, 260–310 bp) (Fig. 5b). The peaks in AT content are positioned in 115–145 bp and 280–310 bp coinciding with the highest peaks of insertion frequency (Fig. 5b). This indicates that sequences where the AT content is higher are more likely to be targets of new insertions as they are more likely to contain the EN cut motif 5′-TTTT/A(A)-3′. In addition, SVA elements show preference on 1–30 bp, which is not explained by the AT content, which could indicate that other factors also weigh in. For L1 elements there is no such detectable preference when comparing the insertion rate to the L1 sequence coverage in the reference genome (Additional Figure S9).

### Replication timing is linked to TE types and target sequence

TE insertions have been shown to prefer both late and early replicating areas depending on their TE type [22, 26, 28, 59]. We wanted to systematically evaluate replication timing [60] of both somatic and germline insertions for all TE types. SVA insertions preferred earlier replicating areas in comparison to other insertions (*p* < 2.2✕10^− 16^, two-sided t-test), while *Alu* and L1 insertions were found more commonly in late replicating areas (Fig. 5c, Additional Table S14). When comparing somatic and germline L1s, we observed that somatic insertions preferred later replication areas than germline polymorphisms (Fig. 5c) (*p* = 8.716✕10^− 5^, two-sided t-test).

As we observed that different TE types preferred different genome annotations (Fig. 5a), we set out to evaluate whether replication timing remained the same for all insertion target annotations (LINE, LTR, SVA, SINE, Simple repeat, Low complexity, Other TE, and unique DNA). As previously reported, we observed that L1 elements tend to be located in late-replicating regions [61]. This pattern was consistent across both somatic and germline events in all target annotations. In contrast, *Alu* events showed a distinct pattern of replication time when the insertion target was their own TE type. Germline *Alu* polymorphisms in reference SINEs are more commonly located in early replicating regions when compared to other *Alus* (*p* < 2.2✕10^− 16^, two-sided t-test) (Additional Figure S10a).

Next, we investigated whether replication timing of target annotations differed from the annotations across the reference genome. To do this, we compared replication timing [60] of overall reference annotations [58] to the replication timing of target areas. We observed that reference SINE elements targeted by TE insertions were more frequently located in early replicating areas than SINEs in the reference genome (*p* = 7.729✕10^− 7^, two-sided t-test) (Additional Figure S10b). On the other hand, reference LINEs and unique DNA showed a preference of late replicating areas when targeted by TE insertions (respectively *p* = 9.413✕10^− 7^, *p* < 2.2✕10^− 16^, two-sided t-test) (Additional Figure S10b). The variability between replication timing, TE type and target sequence shows that TPRT is a complex mechanism likely connected to many factors that we do not yet fully understand.

## Discussion

Transposable elements (TEs) are DNA sequences that can cause insertional mutagenesis and are frequently highly active in several human cancers, including colorectal cancer (CRC). Despite their significance, their impact on both malignant and non-malignant tissues, as well as the differences between germline and somatic insertions, remain poorly understood. Long read sequencing provides a way to analyze the internal structure of TE insertions previously inaccessible to short read sequencing. In this study, we leveraged long-read sequencing to investigate somatic and germline TE by analyzing 56 CRCs and 112 uterine leiomyomas (ULs), aiming to clarify the complexities of both germline and somatic TE insertions and their prevalence in these conditions. As expected, we detected high somatic insertional activity in colorectal cancer. However, despite utilizing various sequencing and detection methods, we did not detect any true somatic TE in ULs. In contrast, uterine corpus endometrioid carcinoma (UCEC) has been shown to exhibit high levels of somatic TE insertions [62]. This difference may be attributable to the malignant nature of UCEC compared to the benign nature of ULs. Additionally, UCEC originates from the endometrium and is an epithelial tumor, whereas ULs arise from mesenchymal tissue, which may further contribute to the observed difference. In fact, osteosarcomas- tumors of mesenchymal origin- have been shown to display infrequent somatic TEs [25, 26, 63]. Interestingly, L1 RNA expression in pancreatic cancer has been linked to loss of *MED12* gene [64] and *MED12* gain-of-function hotspot mutations are driver mutations in ~ 70% of ULs [43, 65]. *MED12* is a unit of Mediator complex, which functions as a bridge from gene-specific regulatory proteins to the basal RNA polymerase II transcription machinery [66]. However, as we observed no insertions in either *MED12* mutant or wildtype tumors, other factors such as tumor malignancy and tissue of origin may contribute.

With the ability to study the inserted sequence as a whole, we examined the length distributions of germline and somatic L1s. In both contexts, the average length of L1s and the proportion of full-length L1s were in concordance with previous reports [23, 46, 67]. As expected, 5′ truncation in germline L1s is less common than in somatic, and it takes place later leading to longer insertions [23]. Additionally, we identified higher insertion length variance in germline insertions than in somatic insertions.

A recent report showcased somatic mosaicism in normal colorectal epithelium [22]. Interestingly, the average length of somatic insertions in our study was similar to those reported in normal colon, and shorter than those in tumors. This could suggest that the bulk of somatic insertions we detect occurred early in tumorigenesis, consistent with the high clonality required with our relatively modest read depth. Strikingly, PCR validation of 3/13 somatic insertions (from two colorectal cancer patients), revealed a trace of the insertion in the corresponding normal colon not detected in the WGS. This suggests that a subset of the detected insertions had taken place prior to transformation [22]. As somatic subclonal events in the normal tissue, they likely would escape detection by WGS. PCR, however, has been shown to detect subclonal L1 insertions left undetected by 40X Illumina WGS [51]. However, other explanations, such as unspecific amplification or the normal tissue containing tumor cells cannot be excluded, despite a histopathology review.

We detected 5′ inversions in germline L1s with a rate of 18.4%, comparable to what has been reported in the literature [14, 37, 38, 68, 69, 70]. The 5′ inversion rate for somatic insertions was higher (21.9%) than in germline L1 polymorphisms, although not as high as the previously reported rate of ~ 30% [22, 23, 24, 25]. However, this could be explained by the use of different datasets, tissue types, and coverage depth. In addition, insertions with 5′ inversion were shorter in soma than in germline and we saw that the end of noninverted cDNA and the start of inverted cDNA are often in vicinity, as reported previously [14, 48].

Previous research on 5′ inversion has mainly been focused on L1s and PPs [25, 47, 71] however, infrequent 5′ inversions have been reported in somatic *Alus* (0.8%) and SVAs (9%) [26]. Although we did not identify any inverted SVA elements, we discovered a germline *Alu* element with a 5′ inversion, adding evidence that it can also occur in germline *Alu* elements, albeit infrequently. This raises the question about whether the rarity of 5′ inversion in germline and somatic *Alus* is driven by mechanistic factors, negative selection, or a combination of both.

A proposed model for these 5′ inversions is twin priming, where reverse transcription starts at two different sites, resulting in two cDNAs with opposing orientations (noninverted and inverted cDNAs) [14]. We found that in insertions with 5´inversions, the noninverted and inverted cDNAs can share sequence, when part of the L1 RNA is reverse transcribed into both cDNAs as previously reported [14, 48]. This overlap is more common in the germline than in the soma. Interestingly, it has a very similar size distribution with target site duplications, which could suggest a similar mechanism. We investigated whether we could identify EN cut sites, however no additional EN motifs or recurrent patterns were present, consistent with the findings reported in Szak et al. [48].

In addition to truncation and twin priming, we found that somatic L1s more often exhibit EN cut site in comparison to germline L1s, possibly due their evolutionary age, as subsequent mutation can disrupt the hallmarks in germline. Analysis of the target sites of the germline and somatic elements revealed no notable enrichment in their presence in genes and fragile sites, however we identified an enrichment of somatic L1 insertions in unique DNA which was not observed in germline polymorphisms. In addition to unique DNA, somatic L1 elements showed a tendency to insert into reference L1 sequences. This tendency to insert into elements of the same type is even more prominent in germline than in soma. These nested elements have been challenging to detect with short read sequencing, however with long read sequencing, the rates of germline L1s inside reference L1 elements have been similar [38]. The nested elements could arise from recombination, although this is less likely since they all presented at least one hallmark of retrotransposition. Furthermore, a notable distinction is the tendency of germline polymorphisms to lay in micro- and minisatellites (1.5% of all germline TE, 2.9% of germline L1), while somatic L1 insertions hit them only rarely (0.9%). While we do recognize the possible confounding by differential sensitivity to detect subclonal insertions in repetitive regions, these differences can partly be attributed to negative selection in germline TEs disrupting functional non-coding elements, such as gene regulatory regions typically found in non-repetitive DNA.

To further investigate insertions inside reference transposons, we characterized whether there were preferences in the target sequence. We identified insertions clustered in three regions in the *Alu* reference sequence, which partly corresponded with AT-rich areas. *Alu* and L1 polymorphisms clustered only at AT-rich sequences, which are likely to harbor more endonuclease cut sites and polyA-tails making them more prone to more insertions. In addition to the AT-rich areas, SVA polymorphisms were present at the 5′ of *Alu* elements which are not AT-rich, suggesting other mechanisms may be involved.

Both somatic and germline L1 elements were consistently identified in late-replicating regions, regardless of the genomic target. *Alu* elements however, display distinct patterns of replication timing when inserted into TEs of their own type. *Alus* inserted into SINEs are predominantly located in early replicating areas, while the *Alus* targeting other genomic regions are found in late replicating areas.

## Conclusions

In this study, we used long-read sequencing to investigate both somatic and germline TE in CRCs and ULs. The advantages of long-read sequencing include its ability to detect TEs within repeat sequences and to capture complete insertion sequences, allowing for a more detailed exploration of TE transposition and insertion structure.

Our study provides new insights into the distinct behaviors of TEs in malignant and non-malignant tissues. The absence of somatic insertions in clonal but benign ULs, contrasting with their prevalence in CRC, suggest that somatic insertions do seldom occur in nonmalignant cells. Furthermore, we showcased key differences between somatic and germline TEs, including variations in transposon type, insertion length, hallmark rates, and target site preferences, highlighting the distinct patterns of TE insertions in different contexts. While previous work [38] has shown that long-read sequencing can detect insertions within repetitive regions that were previously missed by short-read approaches, our study extends these findings by revealing the intricate relationship between somatic insertions, pre-existing transposable elements, and replication timing.

The ability to comprehensively detect structural characteristics like twin priming and nested elements using long-read sequencing offers a more nuanced understanding of both somatic and germline retrotransposition. However, long-read sequencing is still challenged by homopolymers, and it has a higher per-base error rate than short read sequencing. This combined with the lower coverage of the sequencing results in challenges with detecting the hallmarks of retrotransposition. Still, further research is needed to explore the structural details of processes like twin priming. As the study has shown, long read sequencing can help fill critical gaps in our knowledge and offer new perspectives on the role of TEs in cancer biology and genome instability.

## Methods

### Samples

We utilized 272 samples sequenced with Oxford Nanopore Technologies, consisting of 56 colorectal cancers (CRCs) and corresponding 12 normal colon tissues, 106 Uterine Leiomyomas (UL), and corresponding 96 myometrium samples. The CRC samples and the corresponding normals were fresh frozen tumors or adjacent healthy colon and were obtained from a population based series of 1042 CRCs [72, 73] and a subsequent prospective collection of additional Finnish CRCs. The ULs were fresh frozen tumors harvested after hysterectomy, as described previously [45]. All tumors used in this study had at least 50% tumor percentage as assessed by pathology review.

### DNA extraction

Normal and tumor DNA from CRC patients were extracted using DNA isolation standard methods [74]. Both leiomyomas and the corresponding normal myometrium DNA was extracted utilizing the QIAamp FAST DNA Tissue Kit (Qiagen).

### Nanopore sequencing

Sequencing libraries were prepared following the Genomic DNA by ligation (with SQK-LSK109 in hg38 dataset, with SQK-LSK114 in T2T dataset) protocol from the supplier (Oxford Nanopore Technologies ltd). Both sequencing and base calling were performed on the PromethION platform as described previously [45]. In brief, MinKnow-Live-Base calling was utilized for basecalling, minimap2 [75] (v.2.16;preset: map-ont) for alignment to the GRCh38 reference genome (GCA_000001405.15 excluding alt contigs), and nanostat (v1.1.2) and NanoPlot [76] (v1.20.0) were used to assess data quality.

For a recently collected set of ULs that was used to test new R10 flowcells and the novel reference genome, the ligation protocol was SQK-LSK114 and the reference genome was T2T-CHM13v2.0 [49].

For all of the used samples median N50 was 5,500 [Quartiles 4,800, 6,800], min 800 and max 33,800. Median for whole genome coverage for all the samples was 25 [Quartiles 20, 29], min 10 and max 39.

### Identification of structural variants

We identified structural variants (SV) in our dataset using Sniffles (version 1.0.11) [77] with the following parameters: min read support = 3, min length = 10, num_reads_report = 2, min_seq_size = 1000. The parameters were chosen to maximize sensitivity with a lesser concern for specificity as the TE identification provides stricter filtering. To merge structural variants of the same type, orientation and with breakpoints in close proximity, we utilized Survivor [78] (version 1.0.7). The parameters used were the following: “maximum distance” = 50 bp “number of callers” = 1, and the same SV type and strand (SURVIVOR merge 50 − 1 1 1 1–1).

### TE detection pipeline

We developed a pipeline for detecting and annotating transposons from Oxford Nanopore sequencing data (Additional Methods, https://github.com/panummi/TraDetIONS*).* The pipeline utilizes structural variants detected by Sniffles [77] and merged by Survivor [78]. It proceeds in four steps: SV-selection, insertion polishing, annotation, and somatic filtering (Additional Figure S11). First, SV-selection includes insertions with TE sequence by using mappy (2.17) [75] to align the insertions to a TE sequence database. Here we used the sequence database from UCSC RepeatBrowser [58]. In addition to variants with TE sequence, we select insertions with a polyA-tail as potential orphan transductions (TEs without repetitive sequence) and pseudogenes. Finally, the SV-selection step includes deletions with breakpoints near reference TEs as polymorphic reference elements. In the second step, insertion polishing, we use Racon [79] to create a consensus sequence of the sequencing reads supporting the insertion and the flanking target sequence. The polished sequence is annotated in the third step. Annotations identify the target sequence, TE-sequence and the hallmarks of retrotransposition: polyA-tail, target site duplication and deletion, and endonuclease cut site. In addition, it detects sequence that maps to the reference genome (GRCh38) outside the target sequence, and if that locus is within 3000 bp of the 3′ end of the element in the library of full-length TEs, we consider the insertion a transduction. The library of full-length TEs contain the full length L1 and SVA insertion locations as well as full length L1 and SVA elements present in the reference genome. The annotation also recognizes if the insertion contains sequence of a cDNA from Ensembl version 104 [55] and thus is considered a processed pseudogene. Using information from annotation, TEs transposing with TPRT (L1/*Alu*/SVA/pseudogene) without any hallmarks of TPRT (polyA-tail/EN cut site/TSDs) are removed from the dataset to remove SVs containing TE sequence, as they are most likely not result of a retrotransposition event. As the last step, somatic filtering, we distinguish somatic insertions from germline TEs. It compares positions of TE-related insertions to all insertions including insertions without TE sequence. Insertions in tumors that are not within 10 bp of any other insertions in other samples are classified as somatic insertions. The strict distance cutoff is justified by accurate positioning of the insertion by sequence alignment as demonstrated by recovery of the canonical EN cut site motif (Fig. 2c).

### 5′ inversion detection

5′ inversions in the insertions were detected from annotated somatic L1-insertions and germline L1-polymorphisms and pseudogenes. We called a 5′ inversion event if: (1) the insertion contained two areas mapped to L1 sequence with different orientations or (2) the insertion was annotated to contain L1-sequence and one unique sequence (MAPQ > 10) and they had different orientations or (3) the insertion contained two areas uniquely mapped within 500 bp of each other in opposite orientation. To study the breakpoints of insertions with 5′ inversion, we mapped their sequence to the L1-HS reference sequence if the 5′ inversion was inside the L1 sequence [50].

In Fig. 3b, the densities are estimated with a rectangular kernel with bandwidth 40 bp. The polyA-tail is not included in any of the lengths.

### Manual curation

To estimate the rate of false somatic calls, we manually curated [80] 50 random insertions from both CRCs and ULs with corresponding normal either sequenced by Nanopore or Illumina [81, 82]. The insertions were classified as true somatic, germline, and false positive. True somatic calls were required to display insertions with 3 or more supporting reads in the tumor sample and none in the corresponding normal nanopore sequencing data, or if nanopore sequencing not available for the normal sample, less than 4 discordant reads in the normal Illumina data (≥ 40× depth of coverage). Insertions were classified as germline if we could observe in the corresponding normal at least one supporting nanopore read or at least 4 discordant short Illumina reads (if short read sequenced). Insertions were classified as false positives if there were less than 3 supporting reads in the tumor sample. With this procedure we identified 90% (45/50) of the calls to be true somatic in CRCs from which all were L1 insertions (Additional Table S5). As we observed strong TE type bias in somatic error rates in tumors without corresponding normals, we performed additional somatic filtering (Methods TE type based somatic filtering) and curated an additional 50 somatic calls from the CRC dataset utilizing the same parameters as described above. The final proportion of true somatic calls was estimated to be 96% (Additional Table S5). Screenshots of examples in supplement (Additional Figure S12).

As ULs have no previously reported somatic activity, we examined somatic insertion calls in UL tumors with additional scrutiny. We manually curated 56 insertions we had detected as somatic in 38 UL samples with a corresponding normal sample. All insertions were identified as germline (1 nanopore sequencing read in normal supporting insertion), except for 4 insertions detailed in Additional table S16. 3/4 were called true somatic insertions, but their lack of hallmarks, scarcity of TE content, or unlikely TE content (position) marked them as unlikely retrotransposition events, rather they were most likely other structural variants involving TE sequence. One insertion (S_2036) appeared to be a true TE insertion, however, the coverage for the corresponding normal at the site was only 7 reads, thus we could not with certainty call this a true somatic event.

### TE type based somatic filtering

We performed additional filtering to improve the rate of true somatic calls in CRC samples, of which 89% did not have a corresponding nanopore sequenced normal. We compared the number of unique insertions in normal samples to estimate the rate of false somatic calls in tumor samples without corresponding normals. On average, we identified 14.2 unique insertions for each of the 105 Finnish germline samples. CRC samples without corresponding normal had on average 31.46 somatic insertions per tumor. Based on these numbers we estimated that 45% (14.2 unique germline TEs / 31.46 somatic insertions per tumor) of somatic called insertions in CRC samples without corresponding normal may in fact be unique germline TEs. This may be an overestimate as we detected fewer *Alu*, SVA and ERV insertions from CRCs without corresponding normal than expected from germline samples.

We estimated the false positive rate for each TE type in tumors without corresponding normals as a mean number of corresponding TE insertions in a single germline sample. Out of the 1493 unique germline TEs in 105 samples, 72.9% were *Alu* elements, 17.0% L1 elements, 5.9% were SVAs and 3.1% ERVs. For TE types other than L1, the expected number of germline TEs detected as somatic in 50 CRC tumors without corresponding normal, were higher than the observed number of somatic insertions of that type (for *Alu*, the expected number was 517 and we detected 232, for SVA expected 41, detected 12 and for ERV expected 21 and detected 4). In contrast, for L1 insertions, the expected rate of unique germline TEs detected as somatic was 5.7% (expected 83, detected 1468). Based on these results, most *Alus*, SVAs and ERVs insertions detected as somatic (*n* = 253 insertions) are likely to be germline TEs and were thus excluded from the final set of somatic calls.

### PCR and Sanger validation

To validate the presence of somatic insertions, we performed PCR and Sanger sequencing for 13 insertions in 12 samples. To make the validation simpler the insertions were selected at random from somatic insertions that were not in repetitive sequence and were between 100 and 300 bp in size. We used primers that span the insertion and the target sequence on the 5´ end of the insertion and paired them with primers flanking the insertion. This was done in order to amplify only the insertion sequence while still obtaining the full insertion sequence. PCR primers flanking the insertions were designed with Primer3 (http://primer3.ut.ee) and primers spanning the insertion were designed manually. PCR was run in the same conditions for tumor and corresponding normal, and the number of cycles used are in Additional Table S6. We performed the PCR and Sanger sequencing as previously described [43], except that PCR purification was performed with A’SAP PCR clean-up kit, cat no 80,350 − 2000, ArticZymes Technologies. In addition to using insertion-specific primers, we attempted PCR amplification for the same 13 insertions using primers flanking the insertion sites. This approach was intended to generate both the insertion and reference alleles in tumor samples, and only the reference alleles in normal samples. However, the insertion allele was not detectable on the gel. We suspect this is due to preferential amplification of the reference allele, which may have outcompeted the insertion allele during PCR.

### Analysis of insertions inside reference elements

To examine the relative frequency of different reference genome annotations as insertion targets, we compared the insertion counts from different TEs in the annotations and accounted for how prevalent the annotations are in the reference genome. The annotations were LINE, LTR, SVA, SINE, Simple repeat, Low complexity, Other TE (repeats outside previous classifications) and unique DNA (sequence outside the repeat annotations) [58]. For every TE type in all of the annotations, we counted relative insertion rate, a log2 of (((Insertions hitting target + 1)/(Total insertions + 1))/((Target bp in reference + 1) / (reference bp + 1))). Confidence intervals were counted with R boot function (1.3–28.), *R* = 10,000 [83].

To investigate the distribution of TE insertions inside reference genome TEs, we focused on insertions inside the two most common target TEs: *Alu* and L1. To keep the length of the reference TEs uniform, we selected as targets only subfamilies of *Alu* and L1 with canonical length ± 10% of the mode length for the family (L1: 5531–6761 bp; *Alu*: 281–343 bp). Taking insertions inside these subtypes, we extracted the first strand nick position within the canonical length of TE subtypes [58]. To factor in the unequal coverage in the reference genome of the parts of sequence inside the target TEs, we counted the coverage of the TE sequence of the selected subfamilies in the reference genome. We counted the base composition for every position of subfamilies present in the reference to see whether the AT content influences the distribution of insertions in the reference TEs.

### Graph analysis and detection of supporting reads

To review the rate of rare germline variants detected as somatic events in nanopore data, we constructed a node-labelled sequence graph for each donor from the GRCh38 reference genome and the predicted insertions. Each insertion was represented with three nodes: one for the insertion sequence and two for the approximately 2000 flanking bases of the reference sequence on each side. Another node was inserted for the corresponding part of the reference sequence. Consequently, for each insertion, the resulting graph had a pair of node-disjoint paths, colloquially known as a bubble. Additionally, nodes were added to represent the parts of the reference sequences between the bubbles, as well as to the beginning and to the end of the graph. The graphs were annotated with GRCh38’s contigs as well as the insertion identifiers.

We proceeded by realigning the reads to the graph that represented the corresponding donor using minigraph 0.21-r606 [84]. We then counted the alignments that supported either the insertion or the reference path. An alignment was considered to support the insertion path of a bubble if it overlapped both flank and insertion nodes for at least 50 nucleotides. Similarly, an alignment was considered to support the reference sequence path of a bubble in case it enclosed a span of nucleotides which started 50 bases before the end of the left flanking node and ended 50 bases after the start of the right flanking node.

Additionally, we evaluated the somatic status of our calls with discordant read analysis of Illumina paired end and Nanopore long read data. We counted the number of insert supporting reads in the corresponding normals, either sequenced with Illumina or Nanopore. Of the CRCs used in the study, 54/56 had a corresponding normal sequenced with paired-end Illumina sequencing [81]. With Illumina we detected discordant reads: We detected reads in the normal samples within 100 bp of predicted insertion breakpoint that had the pairs mapped to different chromosomes or more than 600 bp from each other. These discordant reads worked as a proxy for presence of TE events in normal samples. With Nanopore we detected supporting reads with the following criteria: insertion length within factor of two of the original insertion, aligned within 100 bp of the called insertion or reads with soft clip of > 30 bp ending within 10 bp of the called breakpoint. For both methods we counted the number of overall reads in the same window and calculated the rate of supporting reads.

## Electronic supplementary material

Below is the link to the electronic supplementary material.

**Additional File 1**: Additional methods and additional figures.

**Additional File 2**: Sequence details of 5′-inverted *Alu*.

**Additional File 3**: Sequence details of Somatic L1 insertion with sequence from nanopore analysis and Sanger sequencing.

**Additional File 4**: Additional tables.

## Data Availability

The software used for TE detection is available in github https://github.com/panummi/TraDetIONS and in Zenodo (https://zenodo.org/records/13284044) as record of archival.The datasets generated and/or analyzed during the current study are not publicly available due to concerns regarding participant/patient anonymity, but are available from the corresponding author on reasonable request.

## Abbreviations

EN: Endonuclease
ERV: Endogenous retrovirus
CRC: Colorectal cancer
L1: Long interspersed nuclear element-1
ORF: Open reading frame
PP: Processed pseudogene
PP: Polymerase chain reaction
RT: Reverse transcriptase
SINE: Short interspersed nuclear element
SV: Structural variant
SVA: SINE-(VNTR)-Alu
TE: Transposable element
TPRT: Target-primed reverse transcription
TSD: Target site duplication
UCEC: Uterine corpus endometrioid carcinoma
UL: Uterine leiomyoma
VNTR: Variable number tandem repeat
