# Supplementary material for: Structural features of somatic and germline retrotransposition events in humans
Source: Mob DNA. 2025 Apr 22;16:20. doi: 10.1186/s13100-025-00357-w (PMC12016303; doi:10.1186/s13100-025-00357-w)
Supplement: Supplementary file 1 — Additional File 1: Additional methods and additional figures. [file 13100_2025_357_MOESM1_ESM.pdf]

Additional information

Structural features of somatic and germline retrotransposition events in humans

Additional Methods

1. TraDetIONS-pipeline

TraDetIONS is a tool that detects and annotates transposable elements (TE) from Oxford Nanopore Sequencing data. It takes detected and merged structural variants (SV) as its input and analyzes them in four steps: SV-selection, Polishing, Annotation and Somatic filtering (Additional Figure S9). As the output, it produces annotated sequences of germline and somatic insertions consisting of processed pseudogenes (PP), partnered and orphan transductions and solo-TEs. In addition, it detects polymorphic germline TEs that are present in the reference genome but not in the sample thus detected as deletions.

1.1 SV-selection

The first step of TraDetIONS is to select SVs detected by Sniffles and merged with Survivor[1] (detailed in Methods 4. Identification of structural variants) that are potentially TE-related. It selects three types of SVs: insertions with TE-sequence, insertions with a polyA-tail, and deletions. Insertions that contain TE-sequence are selected as potential solo-TEs and partnered transductions. These insertions are mapped to a database of transposable elements sequences[2] with mappy (2.17)[3] with the following parameters: preset= "map-ont", k=11, w=6. Insertions that map to TE sequence and are > 40 bp long are selected. TraDetIONS filters away simple repeats with repeat units smaller than 6 bp and repeat covering at least 90% of the sequence to filter out false positives. Although the insertions are previously merged with Survivor[1], the merging step can leave insertions unmerged. Thus, the insertions detected from multiple samples are re-merged based on their TE type and position. For the re-merging, breakpoints in all samples have to be within 40 bp of each other. Also, TraDetIONS requires that they are of the same subtype (Additional Table S17), they share the same orientation, and the transposon length differs max 50% from each other.

Insertions with a polyA-tail are potential orphan transductions and PPs and they are selected if they contain a sequence of As/Ts detected by sweepline technique (where +1 for match, -3 for mismatch and 5 as the limit) within 10 bp of the end of the inserted sequence. We exclude insertions with TE-sequence from them as they are processed as solo-TEs/partnered transductions.

Deletions are selected as polymorphic reference TEs, if they map to a location in the reference genome overlapping a reference transposon (from: https://genome.ucsc.edu/cgi-bin/hgTables,

excluding repeats from repClasses rRNA scRNA, tRNA, RC, and RC?). The deletion and reference transposons are considered overlapping if abs (TE_start - DEL_start) + abs(TE_end - DEL_end) < 11, where the TE_start and TE_end are the coordinates of the transposon and DEL_start and DEL_end refer to the breakpoints of the deletion. The result, the polymorphic reference TEs are called, and no further processing is done to them, as they are present in the genome.

1.2 Polishing

As the inserted sequence provided by Sniffles is based on the sequence of one read, we utilize Racon[4] to polish the insertion and efficiently utilize all reads supporting the call to obtain an accurate consensus insertion sequence. This step includes selecting reads supporting the insertion and selecting a template sequence followed by three iterations of polishing the sequence.

TraDetIONS selects reads supporting the call to utilize their sequence in the insertion polishing step. From the original alignment files, reads with the following criteria are selected: insertion length within factor of two of the original insertion, aligned within 200 bp of the called insertion or reads with soft clip of > 30bp ending within 10bp of the called breakpoint. These reads are referenced as supporting reads. A read with the median insertion length is selected among the supporting reads as the representative read to be utilized as a template sequence in sequence polishing.

After the supporting reads selection, the reads are mapped to a template sequence. Original template sequence is either the insertion sequence provided by Sniffles, or if the polishing is not successful, a representative read selected among the supporting reads. To include the sequence flanking the insertions, TraDetIONS extracts 2000 bp of target sequence from both ends of the

insertion. When using insertion sequence, it extracts the sequence from the reference genome and with representative read, from the read itself.

The supporting reads mapped to template sequence are given as input for Racon. The output of Racon is a consensus sequence of the reads. It is again used as a new template where the reads are realigned and re-input it to Racon. This step is repeated in total three times to improve the quality of the consensus sequence. After three polishing cycles, the annotation is performed for the consensus sequence and used to evaluate the success of the polishing step. If the annotation does not recognize a TE sequence, or an insertion in case of orphan transductions and PPs, polishing is redone using the representative read as the template.

1.3 Annotation

The annotation step is done to a consensus sequence containing the insertion sequence and a total of 4000 bp of flanking sequence. Each polished sequence is further annotated with the presence of the following: target, insertion, non-target, transposon, polyA/polyT, target site duplication/deletion, endonuclease cut site, and pseudogene.

A region of the polished sequence is annotated as target if it maps to the reference genome within 3000 bp from the insertion breakpoint called by Sniffles. Insertion refers to sequences longer than 20 bp not recognized as target. Non-target area annotation refers to sequences mapping to the reference genome, but not in the target area. If the mapping to the reference genome results in multiple non-target areas near each other, the areas within 500 bp of each other are merged together. Transposons contain the sequence mapping to the consensus transposon sequence[2]. PolyA and polyT are annotated if a stretch of A or T bases is detected by using sweep line technique (+1 for match, -3 for mismatch and 5 as the limit). Target site duplication

or deletion is annotated when there is an overlap or a gap between target sequences on both sides of the insertion of size 5-25 bp. Endonuclease cut site is annotated when sequence within 10bp of the insertion breakpoint maps to any of the following motifs with the insertion breakpoint where the dash line is marked (TTTT/A, TTTT/G, TTTC/A).

The pseudogene annotation is used for polished insertions with a polyA-tail. The polished sequence is mapped to cDNA sequences Ensembl version 104[5]. The transcript with the best mapping quality is selected and if more than one had the best MAPQ, the transcript with the longest part mapping is selected. Additionally, the transcript with the best alignment score is selected and if the two transcripts from different annotations do not come from the same genes, we consider both transcripts as the source genes. All the insertions that are mapped to a cDNA are merged together if they are within 40 bp of each other. The merged insertions are re-polished and annotated as previously described (section 1.2 “Polishing” and 1.3 “Annotation”, Additional Methods). Candidate pseudogene insertions are filtered away if any of the following criteria is fulfilled: both breakpoints of the pseudogene and the annotated insertion are more than 40 bp from each other, the pseudogene overlaps with retrotransposon annotation with more than 10% of the pseudogene’s length, the MAPQ for the cDNA is less than 60, the annotation does not recognize any target sequence or the insertion is smaller than 400 bp.

The insertion is called transduction if the non-target area is mapped within 3 kbp of the 3′ end of an L1 from a library of TEs with a MAPQ>0. The library contains full-length (5700-6700 bp) L1 insertions from somatic transposon insertions, and germline transposons from our detection and from the reference genome. If the insertion is among insertions that contain TE sequence, the source transposon is required to be the same type with either the transposons detected with SV-selection or the output from the polished sequence. These transductions are called partnered

transductions. The transductions without TE sequence, with a polyA-tail, are called orphan transductions. For them we required max 150 bp overlap with the source transposon as orphan transductions should not have TE sequence.

All L1/*Alu/*SVA/orphan transduction/PP insertion sequences are filtered out, if they do not have any hallmarks of retrotransposition annotated (polyA-tail/TSD/endonuclease cut site). This is to avoid SV events containing TE-sequence that are unrelated to transposition.

1.4 Somatic filtering

Any insertion called in a tumor sample, however, not in any normal or other tumor sample, is classified as somatic. Additionally, somatic insertions in close proximity (within 10 bp) with an insertion in a normal sample (including non-transposable elements insertions) are filtered away to avoid the presence of false somatic calls.

For PPs, a visualization with BasePlayer[6] is performed to validate the calls. If there are no signs of an insertion, it is filtered out, and the insertions are called germline if their corresponding normal short read WGS contains the same insertion[7].

2. Comparison with prior work

2.1 Long read sequencing tools

Before TraDetIONS, different tools with different emphasis had been created to study TEs in long read sequencing data (Additional Table S18). LoRTIS (Long-read transposon insertion

sequencing software suite) focuses on insertion site sequencing[8], TrEMOLO (Transposable Element MOnitoring with LOng-reads) detects insertions and performs population allele frequency estimation[9]. An early tool to detect TEs from long read data, LoRTE (Long Read Transposable Element) was only compatible with PacBio sequencing[10], but recent tools including TransposonUltimate[11], xTea (x-Transposable element analyzer)[12], TLDR (transposons from long DNA reads)[13], PALMER (Pre-mAsking Long reads for Mobile Element inseRtion)[14,15] Nanomonsv[16], and GraffiTE[17] are capable to process both PacBio and Nanopore data. The pre-existing tools show tendency for either somatic insertions or germline polymorphisms, however, TraDetIONS can readily detect both somatic and germline TEs, as well as polymorphic reference TEs (Additional table S18).

2.2 Performance comparison between TraDetIONS and xTea

To evaluate TraDetIONS performance, we compared it with a published tool xTea[12]. First, we analyzed the Genome in a Bottle sample HG002[18] that has been previously benchmarked and tested with xTea. We were able to estimate the sensitivity and specificity in this data set by comparing the benchmarked *Alu*, L1 and SVA calls containing hallmarks of retrotransposition to TraDetIONS and xTea calls from the same sample. TraDetIONS was able to detect 89% of *Alu*, L1 and SVA calls, and xTea detected 96% (Additional table S2). TraDetIONS called additional 15% of TEs not present in the benchmarked HG002 dataset, while xTea called 32% additional TEs (Additional table S2). As the additional TEs were not present in the benchmarked data set and a minority of them had hallmarks of retrotransposition, these calls are likely to be false positives or recombination events.

To further evaluate the performance of TraDetIONS, we called retrotransposition insertions from four CRCs with both xTea and TraDetIONS. We identified on average 1091 TEs per sample that were detected by both TraDetIONS and xTea. TraDetIONS detected on average 214 insertions not detected by xTea, while xTea detected on average 616 insertions not detected by TraDetIONS. Visual inspection of random 100 insertions that were detected by only one of the TE callers showed that 35/50 insertions detected by only TraDetIONS and 23/50 of insertions detected only by xTea showed any hallmark of retrotransposition (Additional table S3). The comparison between TraDetIONS and xTea with both HG002 and our CRC samples revealed that the two callers mainly detect the same events with xTea being more sensitive while TraDetIONS is more specific.

To evaluate the performance of Nanopore sequencing and TraDetIONS against short read sequencing, we compared the somatic insertion calls from 50 tumors that had both nanopore and Illumina paired-end sequenced data[7]. Short-read sequence calling was performed with xTea case control mode (--case_ctrl) and with default parameters[12]. The main differences between Nanopore and Illumina data are the read length (~6kbp vs. 150bp), read coverage (read depth was ≥40× in the Illumina data), and molecular sampling, with Illumina data sampling much more molecules even with equal read depth. In addition, all the Illumina sequenced samples had corresponding normals in the calling. In total the 50 tumors had 2944 somatic insertions detected with at least one of the callers. As expected, the short read sequencing produced more somatic calls, with 1531/2944 were detected only with short-

read sequencing. This was expected as the sequencing coverage and the number of sampled molecules was much higher with short reads sequencing and the somatic insertions often are subclonal[19]. Both techniques detected 1050 shared insertions (36%), and TraDetIONS detected the remaining 363/2944 insertions. All but two (0.06%) of the Nanopore only calls occurred in a reference repeat highlighting the benefit of long read sequencing in probing the unexplored highly repetitive genomic regions.

Computational issues with xTea

We managed to successfully process only 41 samples with xTea out of 112 UL tumors attempted. This already required modifying the xTea source code to implement file name hashing to avoid trashing our cluster file system with a directory with >100 000 files in it. Ultimately the failed analyses ended up in an infinite loop, apparent from a single candidate insertion that did not show progress after a full week of computation. This could have been alleviated by manually killing certain subprocesses or by further modifying xTea source code by adding a timeout to subprocess call, but we deemed that to be out of scope for this manuscript.

Additional Notes on PCR

In the validation of somatic insertions with PCR, three insertions produced bands in the corresponding normal, although they were not present in the WGS. We examined and excluded the possibility of contamination with the following steps for all the three insertions. Multiple controls of water were run with all gels. A redilution of primer and DNA stocks were produced and used in subsequent PCRs. And finally, multiple primer pairs were used in following PCRs producing the same result, a faint band in the normal.

After the detection of the bands in the normal samples, we applied additional scrutiny for the tissues in question. For the tumor s1708_14_TK and normal s1078_14_N, no tissue was obtainable for future validations (contains insertion S_1745). However, for samples c929_1_T and c929_N tissue was available (contains insertions S_629 and S_1729). After the PCR, both tissues were cut and microscope slides were made of the tissue. Senior clinical pathologist (A.R.) examined the histology of the normal, concluding that the normal slide does not contain tumor tissue. Both the tumor and normal were re-extracted, and new extractions were used in the PCR.

Additional Figures

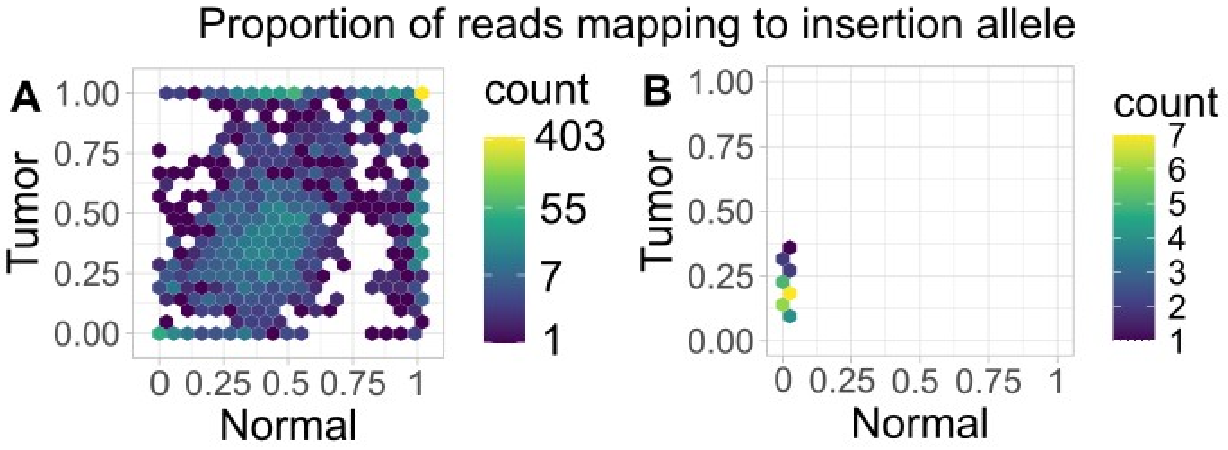

Additional figure S1: The ratio of reads mapping to insertion allele in corresponding tumor and normal with A) germline L1 elements and B) somatic L1 elements. The analysis was done for all the detected L1 elements in six patients with tumor and normal both Nanopore sequenced.

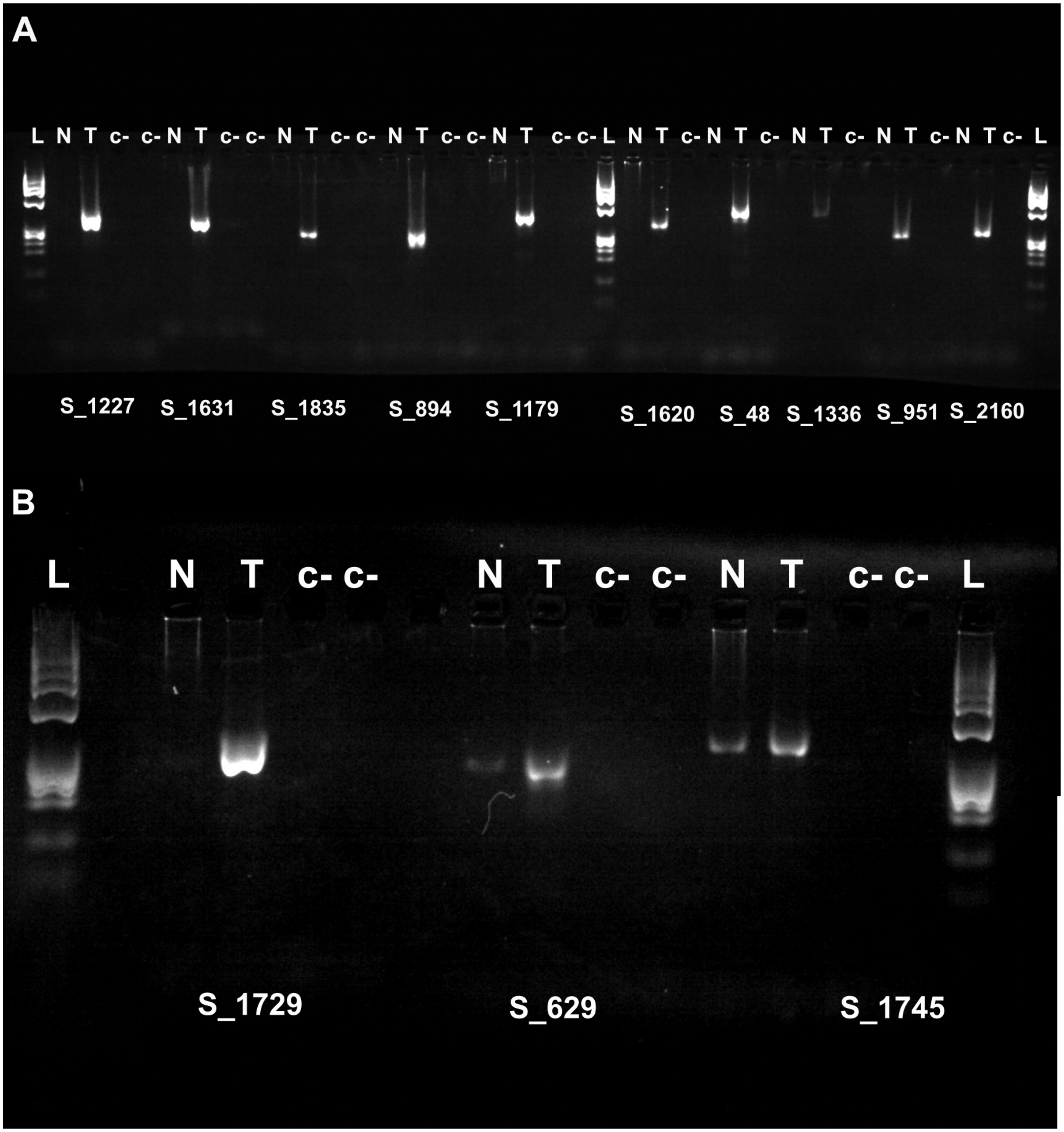

Additional figure S2: Agarose gel (3%) of A) 10 somatic L1 insertions first in tumor and corresponding normal run with the same conditions B) 3 somatic insertions with a faint band in the corresponding normal run with the same conditions. Annotation key: L (ladder; phi174 DNA-HaeIII marker), N (normal), T (tumor), c- (negative control, water). The gel ran with 1h at 50 V, followed by 1 h 30 min on 80 V.

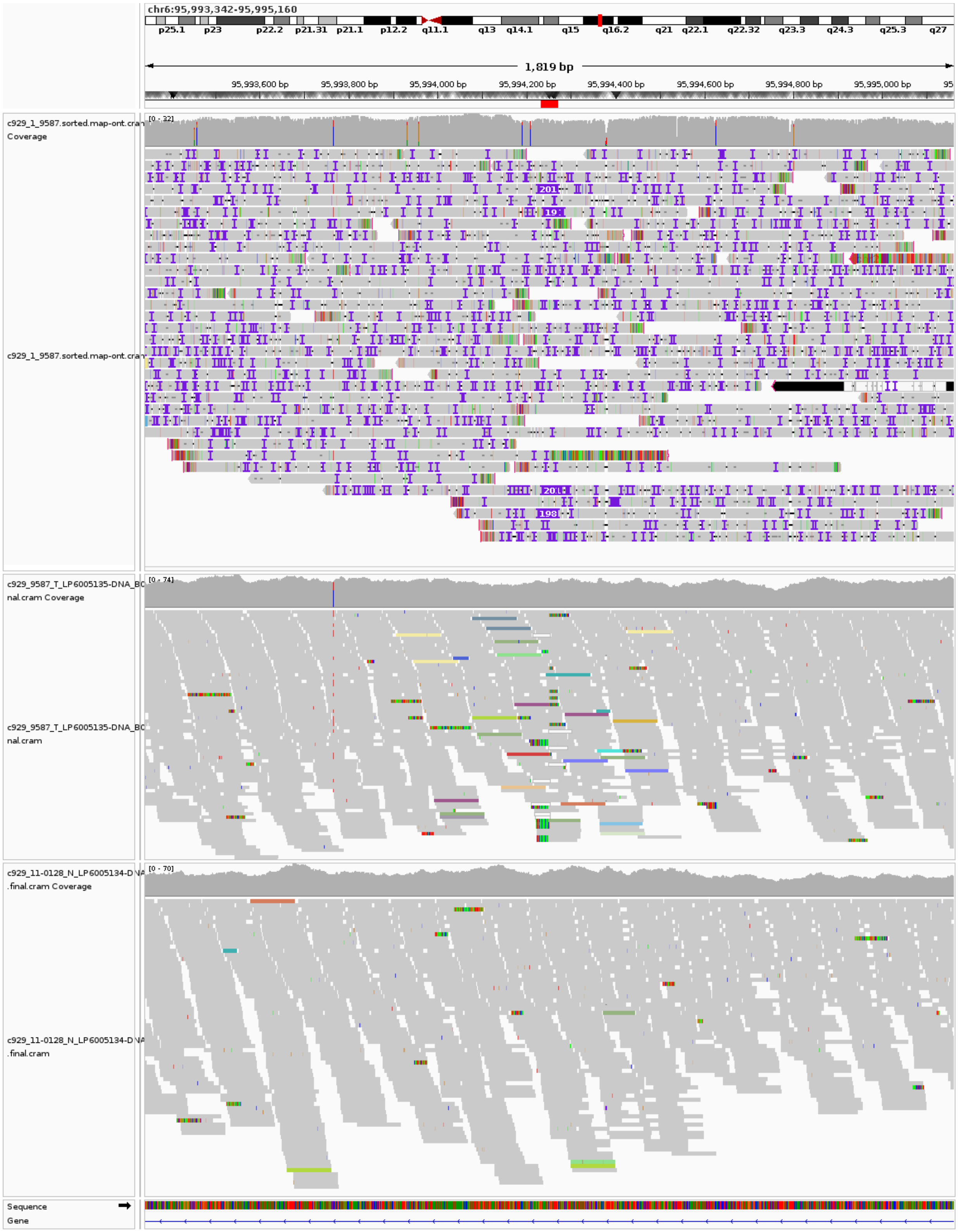
chr6:95,993,342-95,995,160
p25.1 p23 p22.2 p21.31 p21.1 p12.2 q11.1 q13 q14.1 q15 q16.2 q21 q22.1 q22.32 q23.3 q24.3 q25.3 q27
1,819 bp
95,993,600 bp 95,993,800 bp 95,994,000 bp 95,994,200 bp 95,994,400 bp 95,994,600 bp 95,994,800 bp 95,995,000 bp
c929_1_9587.sorted.map-ont.cram Coverage
[0 - 32]
c929_1_9587.sorted.map-ont.cram
c929_9587_T_LP6005135-DNA_B0 nal.cram Coverage
[0 - 74]
c929_9587_T_LP6005135-DNA_B0 nal.cram
c929_11-0128_N_LP6005134-DNA .final.cram Coverage
[0 - 70]
c929_11-0128_N_LP6005134-DNA .final.cram
Sequence
Gene

Additional figure S3: IGV screenshot at somatic L1 insertion at chr6:95994250-95994253 (red bar). Top panel: Nanopore reads from tumor contain ~200bp insertions and clipped alignments at read ends. Middle panel: Illumina short reads from tumor showing clipped reads and multiple reads with mate mapping to other chromosome/location. Bottom panel: Illumina short reads from matching normal, showing scattered unpaired reads indistinguishable from typical genomic regions.

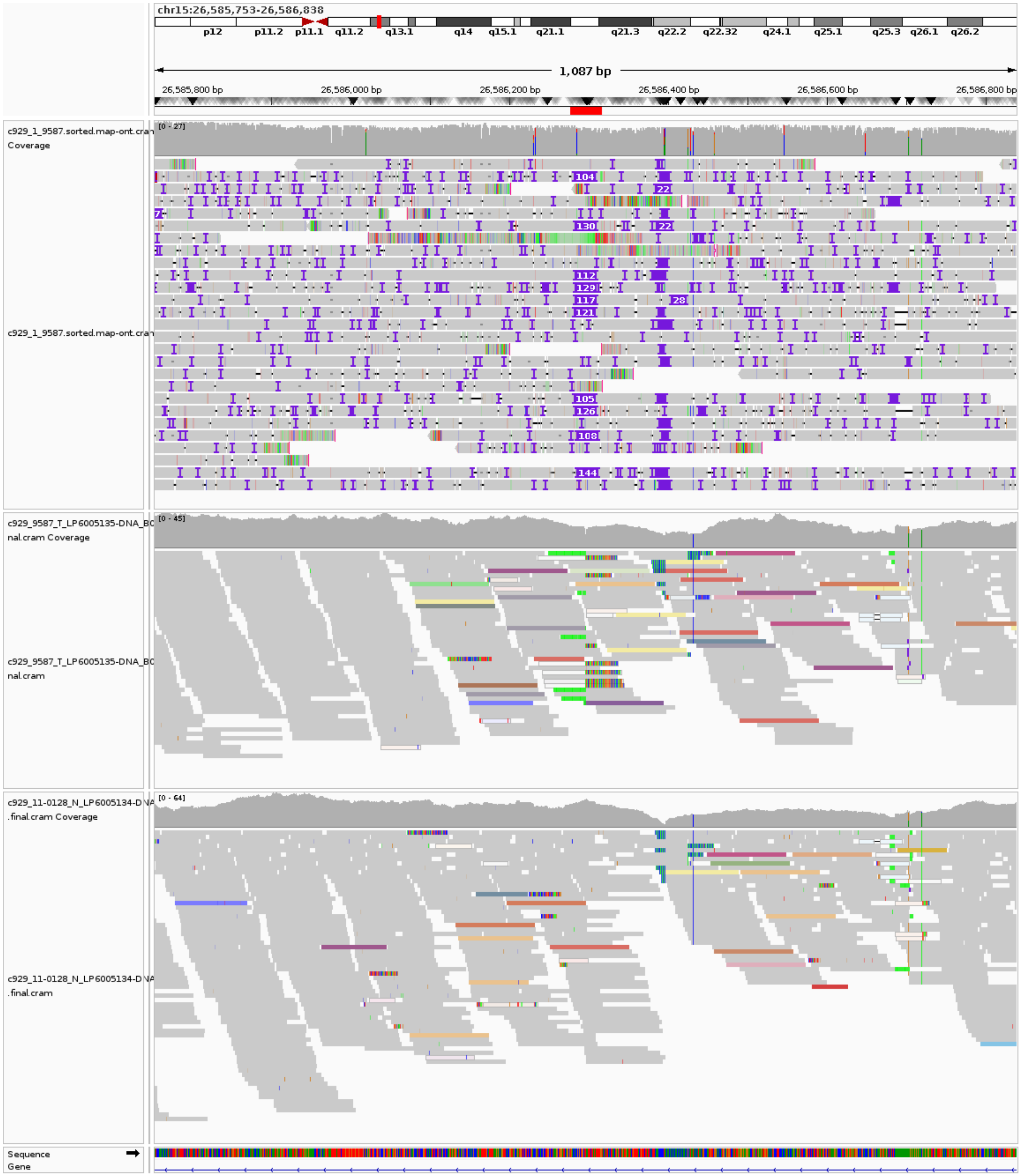

Additional figure S4: IGV screenshot at somatic L1 insertion at chr15:26586297 (red bar). Top panel: Nanopore reads from tumor contain ~120bp insertions and clipped alignments at read ends. Middle panel: Illumina short reads from tumor showing clipped reads and multiple reads with mate mapping to other chromosome/location. Bottom panel: Illumina short reads from

matching normal, showing scattered unpaired reads and expansions of (AC)n and (A)n microsatellites but no signs of TE insertion.

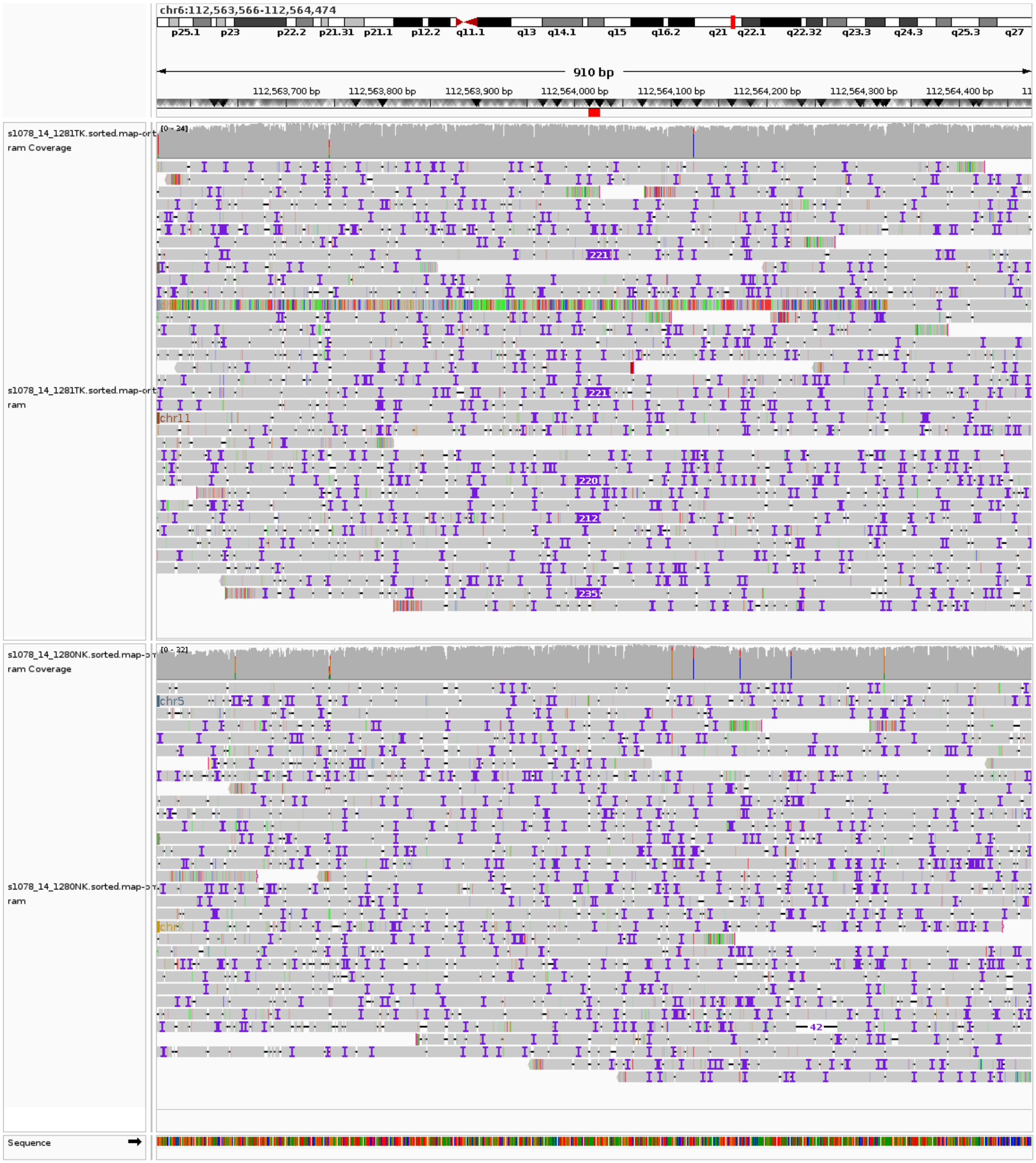

Additional figure S5: IGV screenshot at somatic L1 insertion at chr6:112564015-112564026 (red bar). Top panel: Nanopore reads from tumor contain ~220bp insertions. Middle panel: Nanopore reads from matching normal showing no signs of TE insertion.

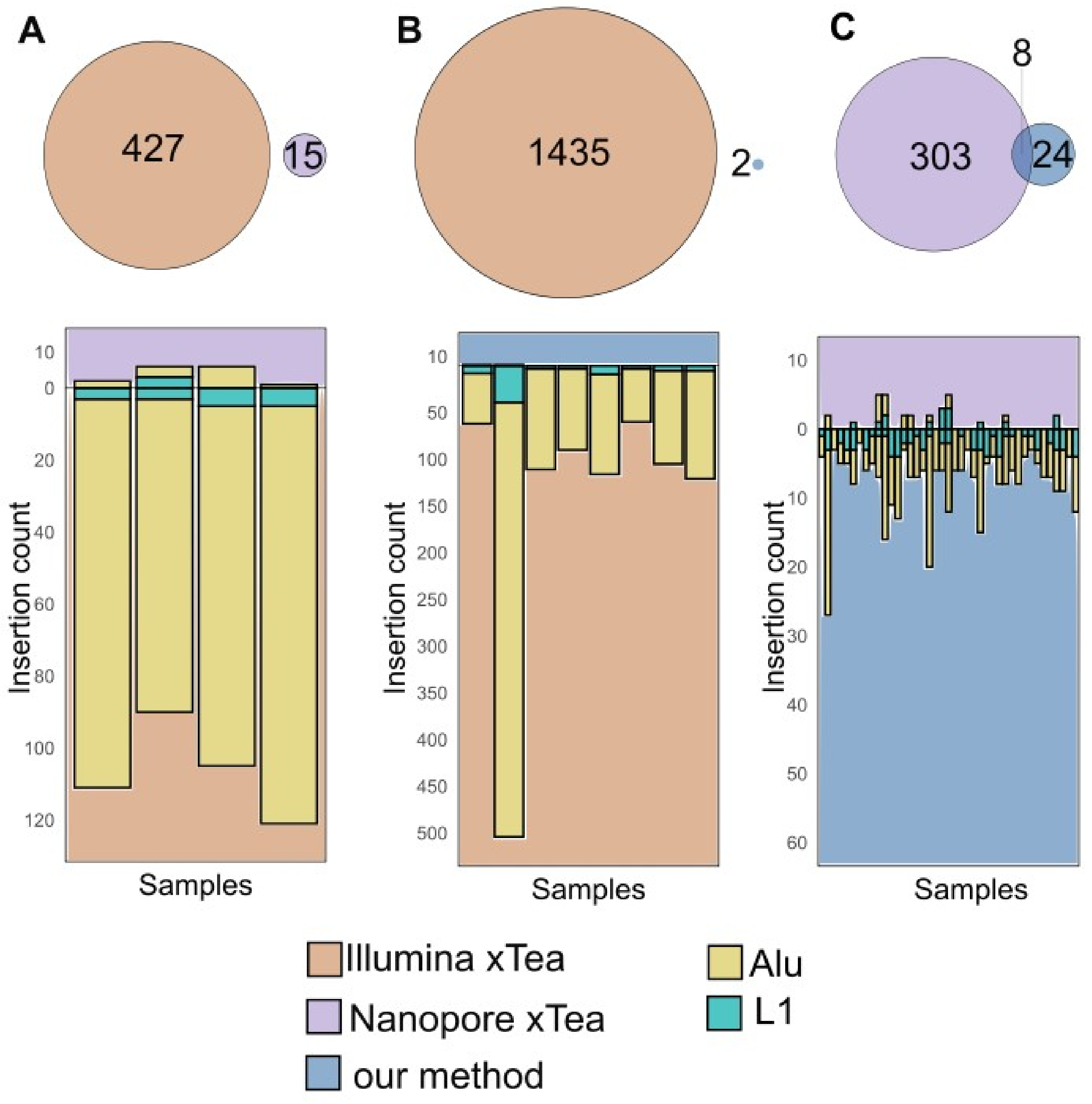

Additional figure S6: Somatic L1 and *Alu* insertions detected from nanopore sequencing with our method and xTea and from Illumina sequencing with xTea. Venn diagrams show the overlap of insertions detected with two methods. Barplots show the distribution and TE type of somatic

insertions between samples in both detections. The number of samples varies between the comparison sets. A) None of the insertions detected by Illumina xTea and Nanopore xTea in four samples were overlapped. B) None of the insertions detected by Illumina xTea and our method in eight samples overlapped. C) Nanopore xTea and our method found only 8 same insertions in 44 samples despite working with the same alignment files. The 8 insertions were later found to be most likely germline based on the number of supporting reads in corresponding normals.

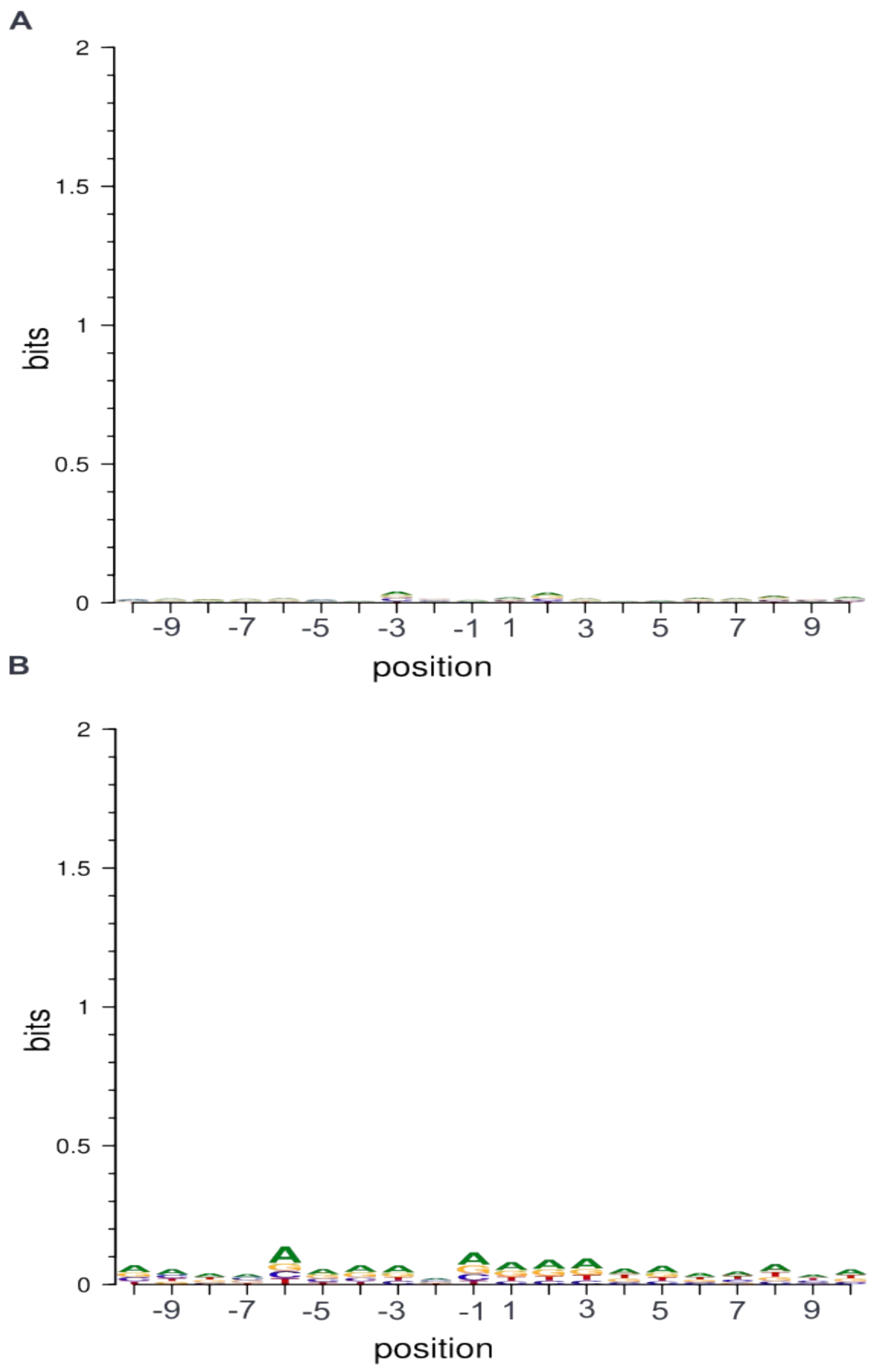

Additional Figure S7. Base composition around 5´ ends of cDNAs without twin priming (A) and with noninverted cDNAs with twin priming (B). Bases 1-10 are extracted from the polished sequences and bases -10- -1 are the bases estimated to have been next in the L1-RNA, extracted

from either L1 sequence where insertion maps to, or in case of transduction, the reference genome.

Additional Figure S4 and Figure 3b produced with SRPlot, http://www.bioinformatics.com.cn/srplot

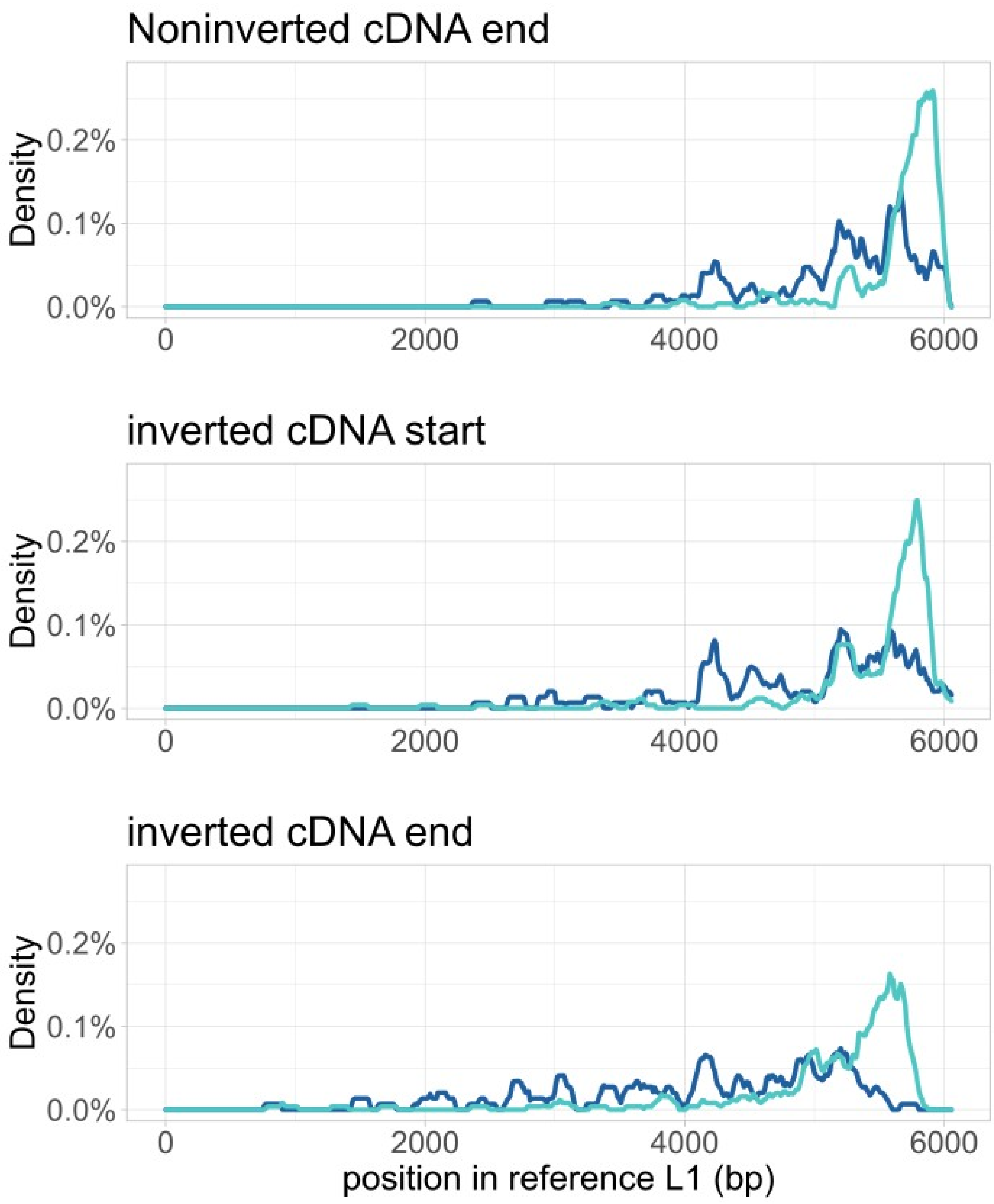

Additional Figure S8. Density of breakpoints in an L1HS sequence with twin priming, rectangular kernel density estimate with bandwidth 40 bp. Y-axis is the frequency of the

breakpoints in the sequence in question. The breakpoint positions within the twin primed L1 are shown in a schematic Figure 3b.

A

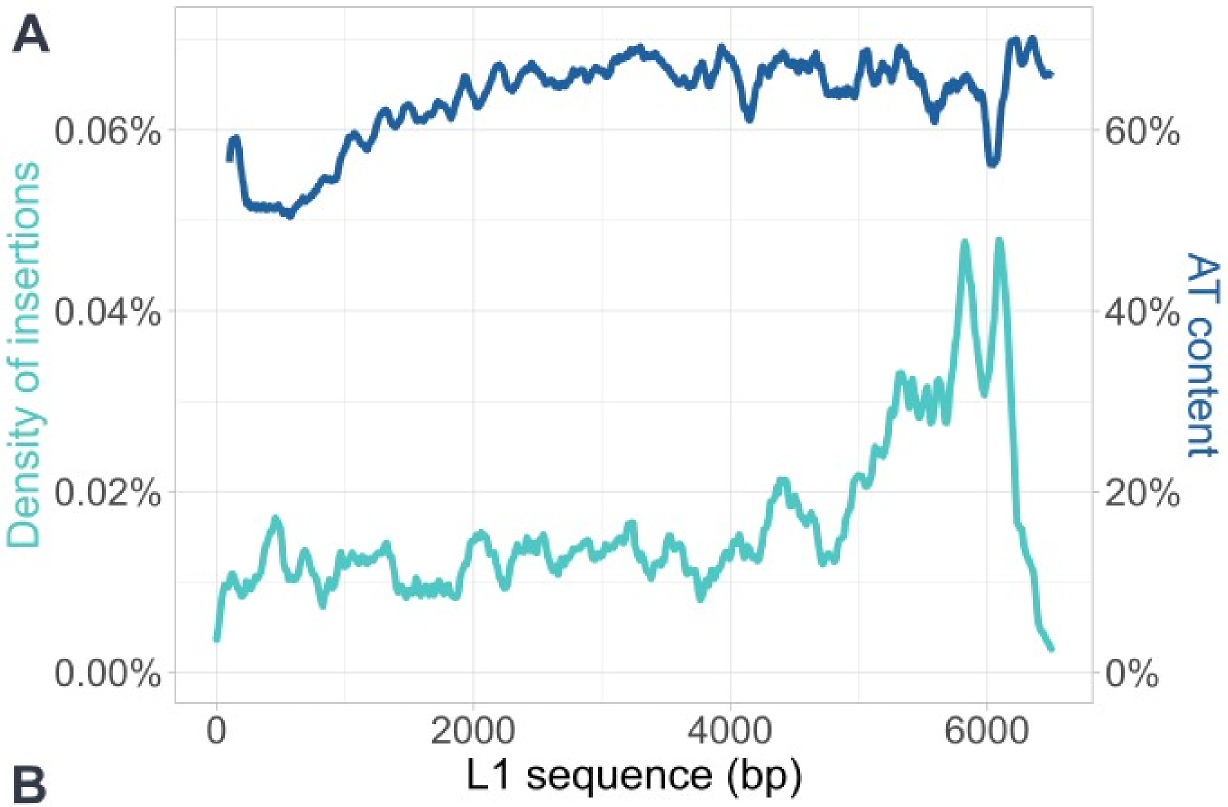

0.06%
0.04%
0.02%
0.00%
Density of insertions
60%
40%
20%
0%
AT content
0
2000
4000
6000
L1 sequence (bp)

B

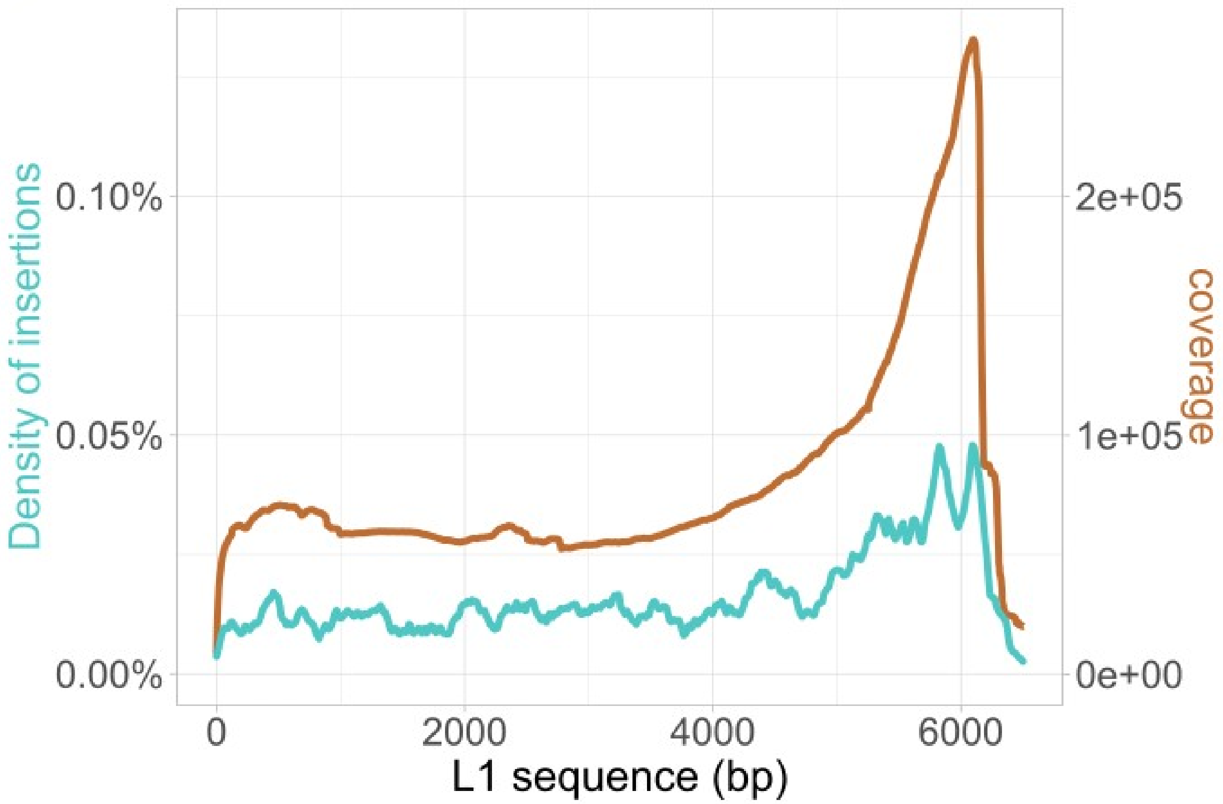

0.10%
0.05%
0.00%
Density of insertions
2e+05
1e+05
0e+00
coverage
0
2000
4000
6000
L1 sequence (bp)

Additional Figure S9. Insertion loci inside reference genome L1 elements. A) AT-content of L1 elements in the reference genome with density of insertions inside L1-elements. The insertions are represented with a rectangular kernel density estimate with bandwidth 10 bp of. A/T-content expresses the AT content in the positions inside reference L1s (smoothed with rolling mean k = 10). B) Coverage/copy number of L1-elements in the reference genome with density of insertions inside L1-elements.

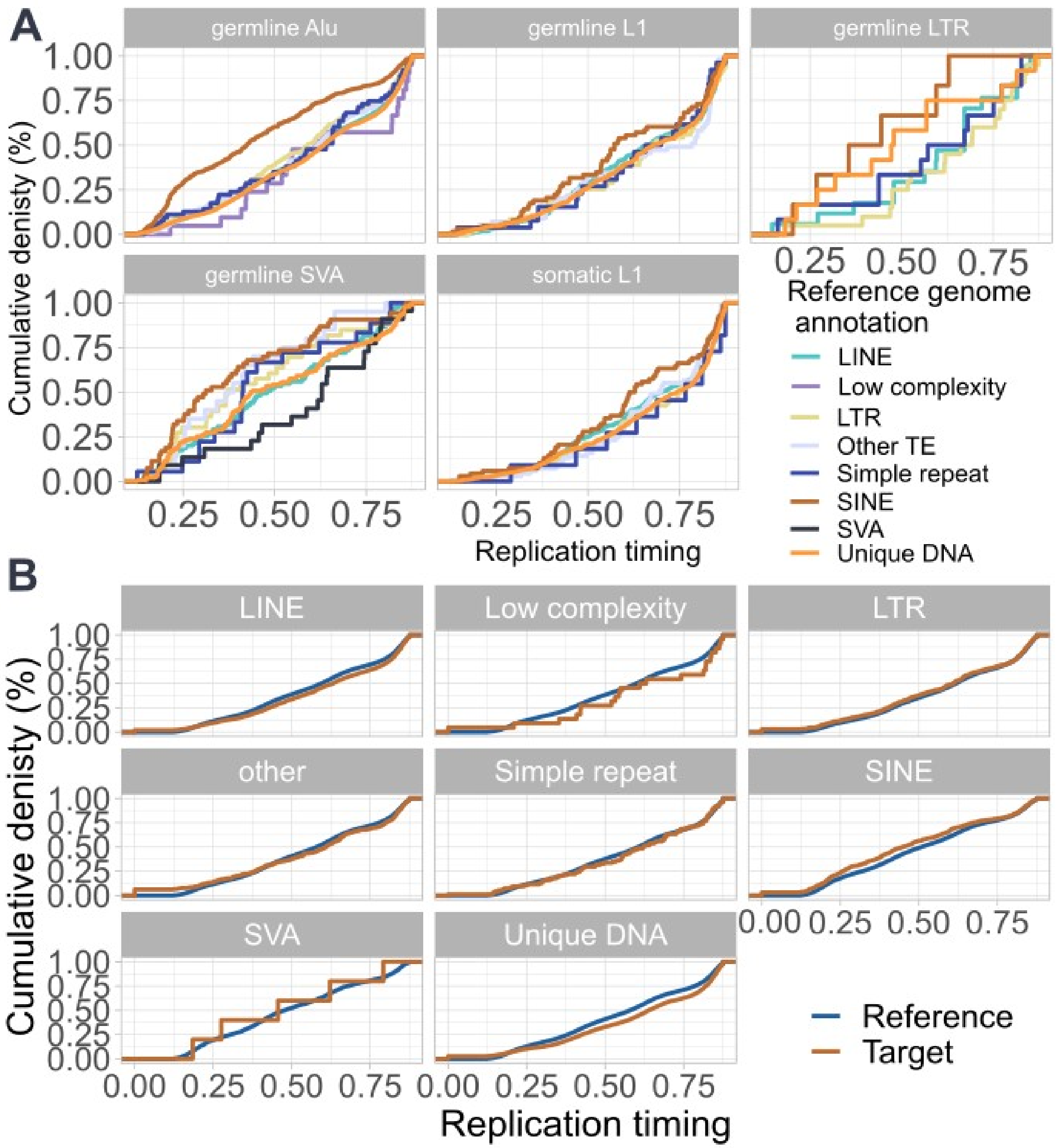

Additional Figure S10. Replication timing in target site annotations A) Cumulative density function of replication timing of target annotations divided by insertion types. B) Cumulative density function of replication timing of target annotations compared to the cumulative density function of replication timing of the same annotations in the whole reference genome.

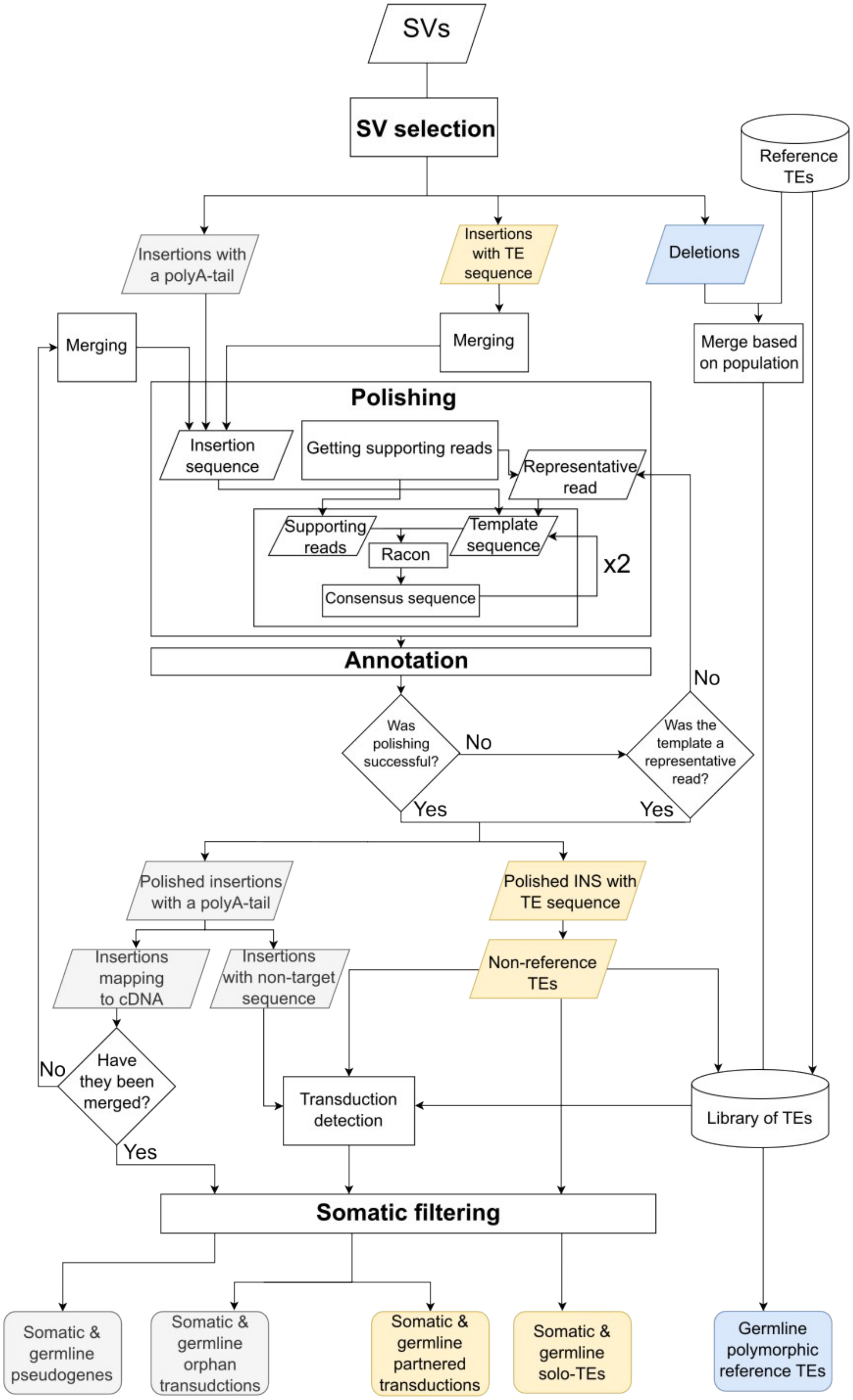
SVs
SV selection
Reference TEs
Insertions with a polyA-tail
Insertions with TE sequence
Deletions
Merging
Merging
Merge based on population
Polishing
Insertion sequence
Getting supporting reads
Representative read
Supporting reads
Template sequence
Racon
x2
Consensus sequence
Annotation
No
Was polishing successful?
No
Was the template a representative read?
Yes
Yes
Polished insertions with a polyA-tail
Polished INS with TE sequence
Insertions mapping to cDNA
Insertions with non-target sequence
Non-reference TEs
No
Have they been merged?
Yes
Transduction detection
Library of TEs
Somatic filtering
Somatic & germline pseudogenes
Somatic & germline orphan transudctions
Somatic & germline partnered transductions
Somatic & germline solo-TEs
Germline polymorphic reference TEs

Additional Figure S11: TraDetIONS (Transposon detection in Oxford Nanopore Sequencing data) detects solo-TE, partnered and orphan transduction, and pseudogene insertions as well as germline polymorphic reference TEs. The pipeline starts with SV selection and continues with polishing and annotating the inserted sequence. As the final step, it performs somatic filtering to distinguish somatic insertions from germline insertions. The different colors represent SV sets that are processed individually in the pipeline to create different sets of results.

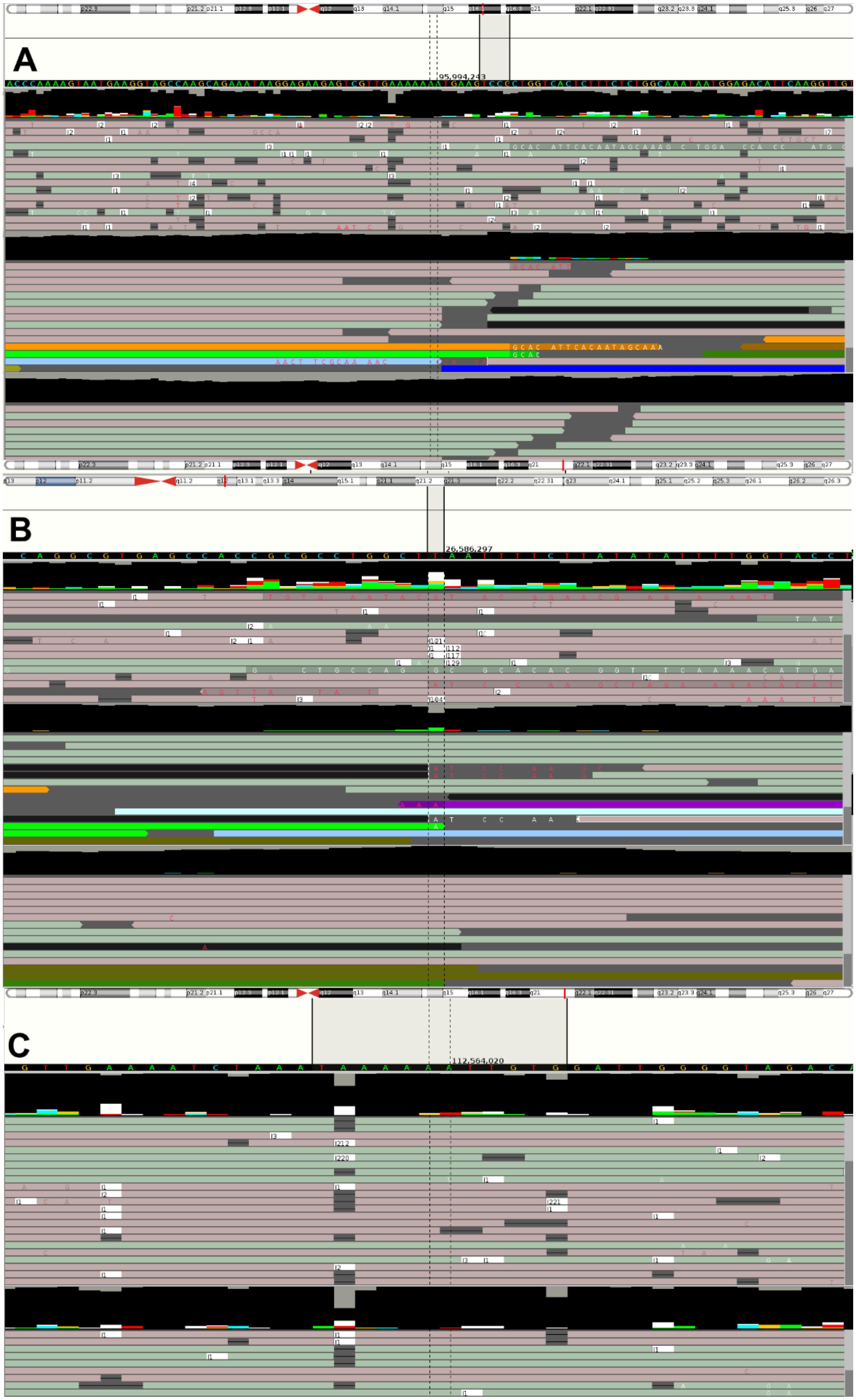
A
95,994,243
B
26,586,297
C
112,564,020

Additional Figure S12. Screenshots of visualization of somatic insertions with BasePlayer. Highest track is Nanopore sequenced tumor, second track Illumina sequenced tumor and lowest Illumina sequenced corresponding normal. A) Insertion S_1121 was classified as germline, as it was seen in the normal sample. Insertions B) S_69 C) S_2009 D) S_1050 were classified as somatic.

Additional tables

Additional Table S1

Table of detected somatic and germline insertions: solo-TEs, partnered and orphan transductions and processed pseudogenes. All insertions have information on the TE type and the hallmarks of retrotransposition including polyA-tail, endonuclease cut site, TSD and twin priming.

Additional Table S2

Comparison of the performance of TraDetIONS and xTea with a benchmarked HG002 sample. The table contains information about number and rate of detected insertions of benchmarked calls and insertions that were not present in the benchmarked dataset.

Additional Table S3

Table of visualization of 50 random insertions detected with only xTea and 50 detected with only TraDetIONS in 4 of our own samples. The table shows the presence of retrotransposition hallmarks based on visual validation.

Additional Table S4

Discordant reads in Illumina sequenced normal samples. Somatic and germline L1 insertions detected from 54 CRC tumors that had a corresponding normal sequenced with Illumina and the number of discordant reads within 100 bp of insertion site detected in aforementioned normals. The table contains the number of discordant reads, the number of all reads in the area, the rate of discordant reads, and whether the samples where insertions were called had a corresponding normal sequence with Nanopore.

Additional Table S5

The table includes the result of the visualization of 50 random somatic insertions in CRC and 50 in UL tumors before excluding other than L1 insertions from the somatic call set (pre_filtering) and 50 CRC insertions after the exclusion (after_filtering tag). The table shows estimated read coverage of the insertions and number of reads supporting the insertion both from the tumor and its corresponding normal sample. If the normal was not sequenced with Nanopore, Illumina sequencing data was used instead. Reads supporting the insertion were defined as reads with an insertion in them or split near the breakpoint in Nanopore data or discordant reads mapping to other TE/polyA-site or split read with the split near the breakpoint in Illumina data. The table also shows the result of the visualization: FALSE, germline or true somatic.

Additional Table S6

The table includes the PCR validation information. The table contains the information of 13 somatic insertions validated with PCR, the predicted sequence, the PCR information, (number of cycles, primers used), the result and Sanger sequence if obtained. In addition, it includes information of the bands in the corresponding normal that were Sanger sequenced.

Additional Table S7

The table includes sequences from tumor and normal samples, where the normal colon exhibited a faint band of the somatic insertion. The table contains the insertion id, tumor and normal sample ids, used primers, and retrieved sequences forward and reverse in both tumor and normal.

Additional Table S8

Number of somatic insertions in CRC tumors after all filtering criteria. This includes somatic solo-L1s, partnered and orphan transductions.

Additional Table S9

Table showcasing the insertions detected as somatic in UL samples with different techniques. The table shows number of insertions, number of samples, rate of L1 from total insertions (L1s and *Alus*), rate of supporting reads in corresponding normal (samples without corresponding normal not included here) and the presence of two hallmarks: polyA-tail and TSD. The methods used to detect somatic insertions in UL are our pipeline used in the manuscript utilizing Nanopore calls, xTea Nanopore and xTea Illumina. As a contrasting data set, the corresponding numbers for CRC somatic calls are present. However, the calls in CRC are more strict, allowing only somatic L1 calls and calls with at minimum one hallmark (TSD/polyA tail/EN cut site).

Additional Table S10

Table of TE and PP events detected from 13 ULs with the recent R10 nanopore and T2T-CHM13v2.0 reference. The table shows the insertion information, TE type and the hallmarks of retrotransposition as well as insertion loci.

Additional Table S11

Table of hallmarks of retrotransposition rates in TE and PP events detected from 13 ULs with the recent R10 nanopore and T2T-CHM13v2.0 reference. The table shows the rates for polyA-tails, TS duplication and deletions and EN cut sites.

Additional Table S12

Table of L1-insertions with an inversion occurring in the L1-sequence. The insertions have been mapped to an L1HS sequence and the breakpoints of noninverted and inverted cDNA presented in the table. The table shows whether there is an overlap between the two cDNAs and contains length of the cDNAs including transduced sequence.

Additional Table S13

Table of somatic and germline processed pseudogene insertions. The table shows the source genes for the insertions and whether the insertions have been previously reported[20–22].

Additional Table S14

Table annotating the insertion site of the detected somatic and germline insertions: solo-TEs, partnered and orphan transductions and processed pseudogenes. The site is annotated for the presence of fragile site, target TE, gene, and DNA replication timing. Furthermore, the genes are annotated with the COSMIC database[23] if they are oncogenes or tumor suppressor genes.

Additional Table S15

Table of somatic and germline insertions in the largest TE groups: *Alu*, L1, ERV, SVA and the number of times they are within different annotations of the reference genome. These numbers are used to estimate the frequency of the TE type in different annotations and its confidence intervals.

Additional Table S16

Table of potentially somatic UL insertions. Insertions in this table are insertions that had no similar event in corresponding normal based on visualization. The table shows the insertion information (TE type, loci), hallmarks of retrotransposition, TE and insertion size, normal coverage and interpretation of the presented insertion.

Additional Table S17

Names of TE subtypes that were used in the merging stage of TraDetIONS. If the TEs in insertions belonged to the same subtype, they were merged together.

Additional Table S18

Comparison of tools created for TE detection from long read sequencing data. In this table there is information on sequencing data and the functions the tools provide, including annotation, somatic filtering, genotyping and transduction/PP detection.

Additional References

1. Jeffares DC, Jolly C, Hoti M, Speed D, Shaw L, Rallis C, et al. Transient structural variations have strong effects on quantitative traits and reproductive isolation in fission yeast. Nat

Commun. 2017;8:1–11.

2. Fernandes JD, Zamudio-Hurtado A, Clawson H, Kent WJ, Haussler D, Salama SR, et al. The UCSC repeat browser allows discovery and visualization of evolutionary conflict across repeat families. Mob DNA. 2020;11:13.

3. Li H. Minimap2: pairwise alignment for nucleotide sequences. Bioinformatics. 2018;34:3094–100.

4. Vaser R, Sović I, Nagarajan N, Šikić M. Fast and accurate de novo genome assembly from long uncorrected reads. Genome Res. 2017;27:737–46.

5. Cunningham F, Allen JE, Allen J, Alvarez-Jarreta J, Amode MR, Armean IM, et al. Ensembl 2022. Nucleic Acids Res. 2021;50:D988–95.

6. Katainen R, Donner I, Cajuso T, Kaasinen E, Palin K, Mäkinen V, et al. Discovery of potential causative mutations in human coding and noncoding genome with the interactive software BasePlayer. Nat Protoc. 2018;13:2580–600.

7. Katainen R, Dave K, Pitkänen E, Palin K, Kivioja T, Välimäki N, et al. CTCF/cohesin-binding sites are frequently mutated in cancer. Nat Genet. 2015;47:818–21.

8. Yasir M, Turner AK, Lott M, Rudder S, Baker D, Bastkowski S, et al. Long-read sequencing for identification of insertion sites in large transposon mutant libraries. Sci Rep. 2022;12:3546.

9. Mohamed M, Sabot F, Varoqui M, Mugat B, Audouin K, Pélisson A, et al. TrEMOLO: accurate transposable element allele frequency estimation using long-read sequencing data combining assembly and mapping-based approaches. Genome Biol. 2023;24:63.

10. Disdero E, Filée J. LoRTE: Detecting transposon-induced genomic variants using low coverage PacBio long read sequences. Mob DNA. 2017;8:5.

11. Riehl K, Riccio C, Miska EA, Hemberg M. TransposonUltimate: software for transposon classification, annotation and detection. Nucleic Acids Res. 2022;50:e64.

12. Chu C, Borges-Monroy R, Viswanadham VV, Lee S, Li H, Lee EA, et al. Comprehensive identification of transposable element insertions using multiple sequencing technologies. Nat Commun. 2021;12:1–12.

13. Ewing AD, Smits N, Sanchez-Luque FJ, Faivre J, Brennan PM, Richardson SR, et al. Nanopore Sequencing Enables Comprehensive Transposable Element Epigenomic Profiling. Mol Cell. 2020;80:915–28.e5.

14. Zhou W, Emery SB, Flasch DA, Wang Y, Kwan KY, Kidd JM, et al. Identification and characterization of occult human-specific LINE-1 insertions using long-read sequencing technology. Nucleic Acids Res. 2020;48:1146–63.

15. McDonald TL, Zhou W, Castro CP, Mumm C, Switzenberg JA, Mills RE, et al. Cas9

targeted enrichment of mobile elements using nanopore sequencing. Nat Commun. 2021;12:1–13.

16. Shiraishi Y, Koya J, Chiba K, Okada A, Arai Y, Saito Y, et al. Precise characterization of somatic complex structural variations from tumor/control paired long-read sequencing data with nanomonsv. Nucleic Acids Res. 2023;51:e74.

17. Groza C, Chen X, Wheeler TJ, Bourque G, Goubert C. A unified framework to analyze transposable element insertion polymorphisms using graph genomes. Nat Commun. 2024;15:8915.

18. Zook JM, Catoe D, McDaniel J, Vang L, Spies N, Sidow A, et al. Extensive sequencing of seven human genomes to characterize benchmark reference materials. Scientific Data. 2016;3:1–26.

19. Pradhan B, Cajuso T, Katainen R, Sulo P, Tanskanen T, Kilpivaara O, et al. Detection of subclonal L1 transductions in colorectal cancer by long-distance inverse-PCR and Nanopore sequencing. Sci Rep. 2017;7:14521.

20. Ewing AD, Ballinger TJ, Earl D, Broad Institute Genome Sequencing and Analysis Program and Platform, Harris CC, Ding L, et al. Retrotransposition of gene transcripts leads to structural variation in mammalian genomes. Genome Biol. 2013;14:R22.

21. Abyzov A, Iskow R, Gokcumen O, Radke DW, Balasubramanian S, Pei B, et al. Analysis of variable retroduplications in human populations suggests coupling of retrotransposition to cell division. Genome Res. 2013;23:2042–52.

22. Feng X, Li H. Higher Rates of Processed Pseudogene Acquisition in Humans and Three Great Apes Revealed by Long-Read Assemblies. Mol Biol Evol. 2021;38:2958–66.

23. Tate JG, Bamford S, Jubb HC, Sondka Z, Beare DM, Bindal N, et al. COSMIC: the Catalogue Of Somatic Mutations In Cancer. Nucleic Acids Res. 2019;47:D941–7.
