# Supplementary material for: Structural features of somatic and germline retrotransposition events in humans
Source: Mob DNA. 2025 Apr 22;16:20. doi: 10.1186/s13100-025-00357-w (PMC12016303; doi:10.1186/s13100-025-00357-w)
Supplement: Supplementary file 2 — Additional File 2: Sequence details of 5′-inverted Alu. [file 13100_2025_357_MOESM2_ESM.pdf]

This file contains the sequence details of an *Alu* insertion with a 5’ inversion in it.

Sequence

Polished consensus sequence of insertion and target sequence. Annotated to show hallmarks and TE sequence. Colors correspond to *AluYb8* reference sequence. See key for meaning:

Key:
target sequence
sequence from *Alu*, inverted, see *AluYb8*-sequence for corresponding sequence
sequence from *Alu*, noninverted, see *AluYb8*-sequence for corresponding sequence
sequence from both inverted and noninverted *AluYb8*, also a microhomology
polyA
TSD
EN-cut site

>G_4753
AGAGTAATTACTTGTTTGGTTGGTGAGTTTTGGGGCTCTATTCTTGACAGAGTCCTTTTGTTAAG
TTGGAGGCTGAGCTTGCTGAGGTATGTTCTGAAAAAGACCATTAATCCATTCTACCTTTCCTGA
AGATTGAGGACAGTAAGGGTATGAGGTTTCAATGAATACCAAGAGCCTGAGAAACTGCTTGGG
TGATTTGACTAGTAAAGGCTAGTCCATTATTAGACTGTATAGAGGTGGGAAGGCCAAACTGAGG
AATTATGTCTGACAGAAGGGAAGAAATAACAATGGTGGCCTTCTCAGACCCTGTCGGGGAAGG
CCTCTACCCATCCAGTGAAAGTGTCTACCCATACCAAGGAGTGTTTTCATTTCCTGACTCGGGGC
ATGTGAGTAAAGTCACTTTGCCAGTCCTGGGCAGGGGTAAATCCCCGAGCTTGATGTGTAGGGA
AGGAGTGGGCTTGAATAATCCCTGAGGAGTAGTAGAATAGTATATGGAACACTGAGAAGTGATT
TCCTTGGGGATAGATTTCCATGATGGAAAGGAAATGAGAGGTTCTAAGAGGCAGTCTAGTGGCT
TGTAACCTACATGGAAGAAGTTATGAAATGACTACAGAATGGAATGGGTCTGTGAGGCTGGAAT
GAGATATTTTCCTTGGTCCAAGAATCATTTGCCTTGTGTGGGAAGAGATTGATAGGTGAAAGTT
TCAGTGGGGGAGTAGGTGGGAGTGACCAGATAAGAAGGAGAAAAACTGCCATGAGGGATAGA
AGTTGGAATGCTAGCTGCTTTTTAGCTACCTTATCAGCATAAGCATTGTCCTAAGCGATGGGATC
TGATGCCTTTTGATGGCTGGTTTTTAGCTACCTTATCAGCATAAGCATTGTCTTAAGCGATGGGG
TCTGATGCCTTTTGGTGGCCTTGGCAGTGAATGACTCCATCTTCCTTTGGAAGTAAAGCCGCCTT
GACAAGAGTTTTTATTAAAGAGGCATTAATAATGGAGGATACTTGTATAGTGAGGAAACATTTT
TCAGCCTATATAACAGCATGGGGTGCAGTATATGGAAAGCATGTTTAGAGTCAGTATAAATATTG
ATGCATAGTCCCTTTGCAAGATTGAGGGCCTGAGTTAAGGCAACAAGTTTGGCTTGCTGAGAG
GTAGCACAGAGGGGCAGAGCGGTAGCCTCAATGATAGACATGGAAGATACTATAGCATAGCCTG
CCTTTGCTGGTGAGTGGCAATTAGGCCTGGTGGAACTGCCATCAATAAACCAAGTGTGATCAGG
GTTAGGAACAGGAAAGAAGGAAATATGAGGAAATGGAGTGAATGTCAGGTGGATCAGAGCGAT
ACAGTCATGTGGGCCAGTTGTGGTATCAGGAATAATGTGGGGGCCAGCCTAAAACAGTAAGGT
CAAGTTGTTTGGACAGAAAGGCTACACAGTGCAGTCCCAGCTCTTGTGTAAAATTTTGACCAC
ACAGCCCTGTACTTGCTGCATGTAATGAAAAGGGTTGCGATGAGTTAAGGAGAGCTAGTGTGGG
AGTAGCTTCTAGGGCTGCTTTTAAGGAATGGAAAGAGGAGTGGTGAAAGGATTTAGGATCTGT
GGGGTCAGCTAGGTTTGCTTTTTGAGTTTATACAATGGTTTAGTCAGGATGGTAAAACTAGATAT
CCAAAGGCAGAAGTACCTAACCATGACTAGGAGGGAAAGGAGTTGTTGTTTTCTAGAAGGGGT
TGGGGTTTGGGAGATTAGCTGGACACAATCGGTAGGGAGAGCACATGTGTTTTCATGAAGAATT
ATGCGAGATAGGTAATGGATGAGAAAGAAATTTGGGCTTGACTGAGGTAATGGGAGCTGTCTGT
GAAGCCTTGCAGCAGTACAGCCCAGGTAATTTGCTGAGCCTAATGGGTGTCAGCATCAGTCCAA
GTGAAAGAGAAGAGAGGCTGGGATGAAGGGTGCGAAGAAATAG*TAAAA*AAGCATGTATTTTT
TAGTAGAGACGGGTTTCACCTTGTTAGCCAGGATGGTCTCGATCTCCTGACCTCATGATCCACCC
GCCTCGGCCTCCCAAAGTGCTGGGATTACAGGCCGTGGCGGGCGCCTGTAGTCCCAGCTACTCG
GGAGGCTGAGGCAGGAGAATGGCGTGAACCGGGAAGCGGAGCTTGCAGTGAGCGAGATTGCG
CCACTGCAGTCCGCAGTCCGACCTGGCGACAGAGCGAGACTCCGTCTC**AAAAAAAAAAAAA**

AAAAAAAAAAAAAAAAAAAAAAAAAAAAAAAAAAAAAAAAAAAAAAAAAAAAAAAA*AA*
***AG*AAA**GCATGTTTGAGATTCAGAACAGACTAATGGGTTATGGAGGGGTTGTGGAGGGAGGTAT
TGAGGATAGGAAAGTATATGGTTTTGACACCAATGGGTGGATAGGCAAGACAATTGGTTGATAA
GGTACAGATCCTGAAATAACCTGTAAGTCTTGTCTGGGTTTTGGACAGGTAAAATGGGGGGAAT
TGTAAGGAGAGTTTATAGGCTTTAAAAGGCCATGCTGTAACAGGCAAGTGATAACGGCTTTAAT
CCTTTGGGGTAAGGGTGATTAGGTTTTAATGGGATGGTAAGAGGTGCATGAATTGTCACCAAGG
AGGGAGTAGAGGTGTCCTATACTTGTGGATTAAAATGGGGAGATACAAGGAGGATGTGAAGGA
GGCTTTGAACTGGGGAAAAGGATGGCAATGAGGTGTGGCTGTAGCCTAGAATAGTCAGGGAAG
CAGATAATTTAGTTAAAATGTCTTGACCTAATAAGGGAGCTGGGCAGGTGGGGATAACTAAAA
AGGAGTGCGTAAAAAGAATGTTGTCCAAGCTGGCATCAGAGTTGGGGAGTTTTAAGAGGTTTA
GAAGCCTGGCTGTCAATATCCACAACAGTTATGGAGGCAAGAGAAACACGCCCTTGAAAAGAA
GGTAATGTGGGGTGGGTAGCCTCCGATTGATTAAGAAGGGTATGGACTCACTTTCCACTGTAAG
AGTTACCTAAAGCATCTGTGATGGTCCAGGAGGCTTCTGAGGTGATTAGGCAGTGTCAGTCTTC
AGCCGCTAAGCCAAGAATATCTGGGAAGGAGTCAGTCAGAGAGCCTTGGGCTAGAGTTCCAGG
GGCTCTAGGAGTGGCTGCCAGGCGAGCTGGACAGTCTGATTCCCAGTGGGGTCCCGCACAGAT
GGGACATGGCTTAGGAGGAATCCTGGGCTGCAGGCATTCCTTGGCCCAGTGGCCAGATTTCTGG
CACTTGAAGCAAGATCCTGAGGGAGGCGGTCCTGTAGGAATGCCTGACCACTGTGGCTTAGGC
ATTTTGAAGTTCTTGTGTGCTGGAGGTGTGGCTGGGTTTTGTCTCATAGCAGAGGCAAGTAATT
TTTACTCTTTCTCTATTATTGTACACCTTGAAGGCAAGGTTAATTAAGTCCTATTGTGGGGTTTGA
GGGCAAGAATCTAATTTTTGGAGCTTTTCTAATGTTGGGAGTGGGTTGGGTAATAAAATACATA
TTGAGAATAAGACTGCCTTCTGCCCTTTCTGGGTCTAGGGCGGTAAACCATCTAAAGTTGTTGC
CAAATGGGCCATGAACTGGGTTGAGTTTCTATATTTGATGAAAAAGAGCCTAAATGCTAACTGA
TTTGGGAAAGGTCAGATAAAGAAAAAGGAGTGTTAACCCTGGCTATGGCTTCAGCTCCAGCCA
CCTTTTTAAGAGGAAATTGTTGGGCAAGTGGGAGAGGGCTAGTCATGGAATGAAACTGTAAGC
CAGACCGGTGTGAGGAGGGGAGGTGATAAAAGGATTATAGGGTGGGGGAACGGAGGCTGAAG
AAGAACTGGGACCTGGCTTGGCCTGGCGAGGAGCAGCTTGGGGAGGAGAGGACAGGTGGGTC
TGTAGAAAAGGAAGATTGAAAAGACTCAGTGATGCTTGGGGTTGGGACTGAAGGACAGGTGG
GAGGGAAAGAAGGAGGATTTGTGATGAGTCGCATTGGGGAACAGAGATTAGGGAGGGACCAA
TGTGTAAAAGAATGCCTGGACATCAGGCATCTCACACCATTTGTCTTTTTGACAAAAAAAATCA
TCCAGGTCTTGTAAAATGGAGGAATCAAAAGTGCCATTTTCTGGCTATTTAGAACCATTATAGA
GTTTGTATTGGGGCCAAGTGGTGTTGCAGAAGAAAATAAGATGCTTAGGTTTTAGGTCAGGTGA
GAGTTGAAGAGGTTTTAAGTTTTTAAGAACACAGGCTAAGGGAGAAGATGCAGGAATGGAGGT
CAGAATGTTGCCCATAGTGAAGGATGTAAGTTTA

***AluYb8*-sequence**

Sequence of reference *AluYb8*, with colored parts correspond to insertion sequence (note that yellow sequence is inverted).

>AluYb8
GGCCGGGCGCGGTGGCTCACGCCTGTAATCCCAGCACTTTGGGAGGCCGAGGCGGGTGGA
TCATGAGGTCAGGAGATCGAGACCATCCTGGCTAACAAGGTGAAACCCCGTCTCTACTAA
AAATACAAAAAATTAGCCGGGCGCGGTGGCGGGCGCCTGTAGTCCCAGCTACTCGGGAGG
CTGAGGCAGGAGAATGGCGTGAACCCGGGAAGCGGAGCTTGCAGTGAGCCGAGATTGCGCCA
CTGCAGTCCGCAGTCCGGCCTGGGCGACAGAGCGAGACTCCGTCTCAAAAAAAAAAAAAAA
AAAAAAAAAAAAAA
