# Supplementary material for: Structural features of somatic and germline retrotransposition events in humans
Source: Mob DNA. 2025 Apr 22;16:20. doi: 10.1186/s13100-025-00357-w (PMC12016303; doi:10.1186/s13100-025-00357-w)
Supplement: Supplementary file 3 — Additional File 3: Sequence details of Somatic L1 insertion with sequence from nanopore analysis and Sanger sequencing. [file 13100_2025_357_MOESM3_ESM.pdf]

This file contains the sequence details of a somatic L1 insertion S_1227 validated with PCR and Sanger sequencing
In graph analysis 3 reads align to insertion graph, while 8 align to reference graph.

Sequence

Polished consensus sequence of insertion and target sequence. Annotated to show hallmarks and TE
sequence. Colors correspond to reference sequence. See key for meaning:

Key:

Target sequence
Insertion sequence
L1 sequence
polyA
Forward primer
Reverse primer
Target site duplication

>S_1227
TTGTATAATTATTCCATTATATATTACAATGTAATAACAGTAGAAATAAGGTGCACAATA
AACGTTATGCCTGTAAATCATCCCCAAACCATGCCCACCCCAAATATATGGAAAAATG
GTCTTCCACGAAACTGGTCCCTGGTGCCAAAAAGGTTGGAGACTGCTGCTTTAAGAG
ACATACATTTGTATGTAATTTAGAATTCTATAGACTGAATGCTCCCTGACACTGGCCAC
TGAGCTAAACCTGAATTATGACTTTTTACCCTTGGCATGGTCTTAGCATGAGTGTAGTA
TCATTCTCAGCCTCTCGACTTGGAGGTTGACCATGTGGCTTGCTTTAACTAACGGTAT
GTAGTCATCCATGACCGTGTGCTTTTCTGAATCTAAGTCTTAAGAGTCTCATGTAATTC
TGTGTATTGCCTTGTGCTTTTGACATCACTATGATAAGAGCATGCCCTGCTGATCTGCT
AATCCAAGGAGGATGACATACATGGAGAGTGGACTAGAACTCACAGACCTGCAGTGT
GGAGAAAAATGGTACATCTAAACCCAACCTGGAGCAGATGACCCCTAGGTGACCTCA
GTTTCATGAAAAAACAGTTAAAATCAGAAGACCTTGTAAAGCTGAGCCCTGCCCTGG
TCAGCCAACTCACAGTTGGCTGATAATGCCTCAGGGCATTAGCAGGTCTATGATTCTT
AATGTAGTTTGATATAAAAGCACACTTTTTGGTGTGGTTTAGCTCCATGTCTCCATCCA
AATCTCATCTTGAATTGTGATCCCTATGTGTCGAGGGAGGGTCCTCATGGGAGATGATT
GGATCATGGGGGCAGTTTCCCCCATGCTGGTCTCATGACAGTGAATGAGTTCTCAAGA
GATCTGGTTGTTTGAAAGTGTGGCACATCTCCTATCTCTCCGTCTCCTGCAGCTATGTA
AGAATTGCTTTGCTTTCTCTTTGCCTTCCACCAGGATTGTAGGTTTCCTGAGGCCTCCC
CCGCCATAAGAACTGTGAGTCAATTAAACCTCTTTTATTTATAAATTACAGTTTCAGGT
AGTTTTCTATACTTGTAGCGTGAAAATGGACTACCCTTTTTTTTTACTTTGATTTTGTTC
GTTCATTTGGCAATAGCCAATTAATACAACTACTTTATTTTTCATAGATATTTTCCTGTG

TTTCTTCTTCTGTCCTTTTGCTAAAATGGGTAATTGATATTAAATAAAGTAAGTTACTAA
GTGTACGTAAAATTACTACAGTGTGAGGACATTACAGTGTACGTACTATGAACAGTAG
ACTAACCAACAGGTCAACTAGGTTCAGTTTATTCCAATTCACAAGGGGAATGCTTTGA
GAGTTTTTGTATTTGTCTGTTTTTTTGTTTTTTTGGACCACCTAGCTTTCTTTTTAGTCT
GGTAATTGAAATCTCTTTTTCTTTTTGATATTTTCTTTGATGTATGAATAACTACTTGGT
GATACCTTAAAAAAGCATGCTCTGAAAAGCCACCCTAAAGCCAAGTATTGAAGCTTTA
GTCTCCTTAAATTTTATTTAAACATTTAGTAACTTTGCTTTATCTGGATAAGCTCTATCT
GAGCAGTTCTCTAAGTGCTGCTCACTGCCGTCAAGTCATTTATAACCCATCAACATTG
CACTTAGTTTTATCTACTCATGTTTTATCTTCTTTAAAGGTAAGGATGCTATTGGGTTGA
ATTCAGTAAGACATAGAAGTGAATTTTCACTGCCATCATAAAATTCTGCCTGGTTCTTA
CTGTGTCATGGCAAACATTTGTGAGATTCTTAGTTTATGTTACTAGTTCAATGTGTACT
CAAAGTCTAACTAAATCACAAAGAATGAAGTTTCAATTCTCTCCCCAGAATAACAGCT
ATGTTGGGAAACTCACAGTCACCTGATTGGAACACAAAGTCTATATTCAATGGCAAA
AGAAACCAAATCACTTAAGTCAGGATAATAAACAATCACAATGTAGAA*GGGGGGATA*
GCATTGGGAGATATACCTAATGCTAGATGACACATTAGTGGGTGCAGTGCACCAGCATGGC
ACATGTATACATATAACTAACCTGCACAATGTGCACATGTACCTAAAACTGAGAGTATAAT ***AAA***
AAAAAATAAAAAAAAAAAAAAAAGAAAAAAAAAAATTAGGTTGTGTTTATATAGTTTTTT
TTAACATCTAGCGAGTGTAAGATGCCAAAGGCAAGCTTAGATAAAAACCACTGTAAA
ACTGATTTCATTATAACACCAGTGTAGAATCCAGGCAATAATTCACTTAATCATGCTCT
ACAAAATCAAGATTGCATGAAGTTCTACTGTGAATGGCTGTCATTACTATTGATTAGAA
TAACTAGATTTAGACAAGTTGCTGAAAGGATAGACTTAAAATAATCAAGTTTCAATAA
CATTTCACTTTTAGAGAAATCTCAGAAAGATATAATCAAGAAATCACTTTCAAATAACT
AGGACTTCATAAATCCCTTTTGAAAAAAATTTATTTTCCAAAATAAATAAAAATAGAA
GAGAATATAAGAGAAAGGTATGTGTACAATATGATCAGGCATTATGAATATATGTGTGT
GTATATATATATATATATATATATATATATATATATATATATATATATGTATACCTTAAATGTGT
GTGTTTTTGTGTATAAAAATGCATCTCTCTTCCCAGTGTCATATCATCTATGGGATGTGG
AAGAGCTAAACTATTATACATGGTAAGAATGATTTATAAATGATGCTATTTTCTAGTTAG
TGATAAAAATGAAAAATATCTGGGAACTTAAATATTTATTTCTACCTTTTTTGACAAATT
GAGACCAAATTCTAGTTATTGAATCAGAACGTCTAATTTAGATGTAATGGAGTATACAA
CTGCCAAGTGTCTTCTACAAGGCAGTGACAGTTACAAGTTCTGTAGACCAGTTGAGT
CATTCTTCTTGTATCAATCTGAGATGACTTAGCAGGAGACCGACCCACACATGCAAAT
TTTAATTATTTTTTCTTTCCTTTATTTTATTTTATTTTATTTATTTATTTATTTTGAGAT
GGAGTCTCCTCTGTCACCAGGCTGGAGTGCAGTGGCACGATCTCGGCTCACTGCAAC
CCCCACCTCCTGGGTTCAAGCGATTCTCTGCCTCAGCCTCCCAAGTAGCTGGGATTAC
AGGAGTGCACCACCACACCTGGCTAATTTTTGTATTTTTAGTAGAGTTGGCATCTCAC
TGTGTTGGCCAGGATGGTCTCTATCTCCTGACCTCGTGATCCACCCACCTTGGCCTCC
CAAAGTGTTGGGATTACACCTGTTAGCCACCGTGCCCAGCCTACAAATTTCTATTTTTA
TTTCATTTGAAAACTCACCAGCAACAAGTGGAGCATATAAAATAAAATGAAGAAACA
ATTTAAAACTACAAATTAATTATGAATAAAGAAAATAACTATGGATGCTTTATAACGTA
AATTCAAGGAAAATGTAGAAAAGTTTTGGAGGTGGTAAATTTTAAAAATGCTTTTTTA

TATATTAATAATAAGTAAAAGCATACCCAGACTGATTTTACCTAGTTCTTGCTGAAACT
CACTGGTCTTCAAGAAGAGTTCTACAAAACTGAGATTTTCAGGGCAGTCTAACAGTT
TTGATTCAAGACAGGAACATGCCTAGATTTTAGCTCCCATGCTAAACTTTATCAAGGTA
ATTTTGCTTTTATCCATTGCTTTTTAATATTAATGTTGTGCAGCAAAATATTTATTTTACA
AACTATTTTCAATATAACTTGTTTGCAAAAGAATGGCTGTATTACTCAATATCTTTAATA
TTTTTAATTAGTTATTAATTTAAAGTTTGTGATATGAAGTTAATAAACATTGAGCAGATT
ATAATGACCTAAATGTAATCTATAGTACGGTAGCTAATATTAGCAATGTTGCTAGACGCT
TTTAAATTAAACTGCATATTCAACTACAGGTTTCAATCATTTCAGAGTAATTTATATTTG
ACATATTCTTCCTCTGAAAACAACTATAAAACCAAGAAAAAATATGTATATGGAGATTC
TTTTCAGGCATTGAACAACAGAAATGCATGACTGAGATTCCTTTAGGGAATGGGCAA
ACATGAAGTGACTCTGTCTCCTTGGCCT

Reference sequence

Reference sequence used in graph analysis
AGAA is the sequence forming the target site duplication in the insertion allele.

AGAACTGTGAGTCAATTAAACCTCTTTTATTTATAAATTACCCAGTTTCAGGTAGTTTT
CTATAGCAGCGTGAAAATGGACTACTACCCTTTTTTTTTTTGCTTTTGATTTTGTTCGTT
TCATTTGGCAATAGCCAATTAATACAACTACTTTATTTTTCATAGATATTTTCCTGTGTTT
CTTCTTCTGTCCTTTTGCTAAAATGGGTAATTGATATTAAATAATTAAGTAAGTTACTAA
GTGTGCCACGTAAAATTACTACAGTGTGAGGACATTACAGTGTACGTACTATGAACAG
TAGACTAACCAACAGGTCAACTAGGTTCAGTTTATTCCAATTCACAAGGGGAATGCTT
TGAGAGTTTTTGTATTTGTCTGTTTTTTTGTTTTTTTTTTGGACCACCTAGCTTTCTTTT
TAGTCTGGTAATTGAAATCTCTTTTTTTTCTTTTTGATATTTTCTTTGATGTATGAATAAC
TACTTGGTGATACCTTAAAAAAGCATGCTCTGAAAAGCCACCTAAAGCCAAGTATTGA
AGCTTTAGTCTCCCTTAAATTTTATTTAAACATTTAGTAACTTTGCTTTATCTGGATAAG
CTCTATCTGGAGCAGTTCTCCTAAGTGCTGCTCACTGCCGTCAAGTCATTTATAACCCA
TCAACATTGCACTTAGTTTTATCTACTCATGTTTTATCTTCTTAAAGGTAAGGATGCTAT
TGGGTTGAATTCAGTAAGACATAGAAGTGAATTTTCACTGCCATCATAAAATTCTGCC
TGGTTCTTACTGTGTCATGGCAAACATTTGTGAGATTCTTAGTTTATGTTACTAGTTCA
ATGTGTACTCAAAGTCTAACTAAATCACAAAGAATGAAGTTTCAATTCTCTCCCCAGA
ATAACAGCTATGTTGGGAAACTCACAGTCACCTGATTGGAACACAAAGTCTATATTCA
ATGGCAAAAGAAACCAAATCACTTAAGTCAGGATAATAAACAATCACAATGTAGAAA
AAATTAGGTTGTGTTACATATAGTTTTTTTTAACATCTAGCGAGGTAAGATGCCAAAGG
CAAGCTTAGATATAAAACCACTGTAAAACTGATTTCACATAACACCAGTGTAGAATCC
AGGCAATAATTCACTTAATCATGCTCTACAAAATCAAGATTGCATGAAGTTCTACTGTG

AATGGCTGTCATTACTATTGATTAGAATAACTAGATTTAGACAAGTTGCTGAAAGGATA
GACTTAAAATAATCAAGTTTCAATAACATTTCACTTTTAGAGAAATCTGTGAAAGATAT
AATCAAGAATATCACTTTCAAGTAACTAGGACTTCATAAATCCCTGAAAAAAATTTATT
TTCCAAAATAAATAAAATAGAAGAGAATATAATAGAGAAGGTATGTGTACAATATGATC
AGGCATTATGAATATATGTGTGTGTATATATATATATATATATATATATATATATATATAT
ATATGTATACCTACGTGTGTGTGTTTTTGTGTATAAAAATGCATCTCTCTTCCCAGTGTC
ATATCTATGGGATGTGGAAGAGCTAAACTATTATACATGGTAAGAATGATTTATAAATG
ATGCTATTTTCTAGTTAGTGATAAAAATGAAAAATATCTGGGAACTTAAATATTTATTTC
TACCTTTTTTGACAAATTGGAGACCAAATTCTAGTTATTGAATCAGAACGTCTAATTTA
GATGTAATGGAGTAACACAACTGCCAAGTGTCTTCTAAGGCAGTGACAGTTACGTTCT
GTAGACCAGTTGAGTCATTCTTCTTGTATCAATCTGAGATGACTTAGCAGGAGACCGA
CCCACACATGCAAATTTTAATTATTTTTTCTTTCCTTTATTTTATTTTATTTTATTTATTTA
TTTATTTATTTTGAGATGGAGTCTCCCTCTGTCACCAGGCTGGAGTGCAGTGGCACGA
TCTCGGCTCACTGCAACCCCCACCTCCTGGGTTCAAGCGATTCTCCTGCCTCAGCCTC
CCAAGTAGCTGGGATTACAGGAGTGCACCACCACACCTGGCTAATTTTTGTATTTTTA
GTAGAGTTGGCATCTCACTGTGTTGGCCAGGATGGTCTCTATCTCCTGACCTCGTGAT
CCACCCACCTTGGCCTCCCAAAGTGTTGGGATTACACCTGTTAGCCACCGTGCCCAG
CCTACAAATTTCTATTTTTATTTCATTTGAAAACTCACCAGCAACAAGTGGAGCATATA
AAATAAAATGAAGAAACAATTTAAACTACAAATTAATTATGAATAAAGAAAATAACTA
TGGATGCTTTATAACGTAAATTCAAGGAAAATGTAGAAAGTTTTGGAGGTGGTAAATT
TTAAAATGCTTTTTTTATATATTAATAATAAGTAAAAGCATACCCAGACTGATTTTACCT
AGTTCTTGCTGAAACTCACTGGTCTTCAAGAAGAGTTCTACAGGAAACTGAGATTTT
CAGGGCAGTCTAACAGTTTTGATTCAAGACAGGAACATGCCTAGATTTTAGCTCCCAT
GCTAAACTTTATCAAGGTAATTTTGCTTTTATCCATTGCTTTTTAATATTAATGTTGTGC
AGCAAAATATTTATTTTACAAACTATTTTCAATATAACTTGTTTGCAAAAGAATGGCTG
TATTACTCAATATCTTTAATATTTTTAATTAGTTATTAATTTAAAGTTTGTGATATGAAGT
TAATAAACATTGAGCAGATTATAATGACCTAAATGTAATCTATAGTACGGTAGCTAATAT
TAGCAATGTTGCTAGACGCTTTTAAATTAAACTGCATATTCAACTACAGGTTTCAATCA
TTTCAGAGTAATTTATATTTGACATATTCTTCCTCTGAAAACAACTATAAAACCAAGAA
AAAATATGTATATGGAGATTCTTTTCAGGCATTGAACAACAGAAATGCATGACTGAGA
TTCC

Primers

S1227_over_Fb
AACAATCACAATGTAGAAGGGG
S1227_out_Ra

TGCCTGGATTCTACACTGGT

ACCAGTGTAGAATCCAGGCA (reverse complement)

Sanger sequence

Sequence obtained from PCR product in both forward and reverse, coloured parts correspond to polished sequence

Forward:
AATGCTAGATGACACATTAGTGGGTGCAGTGCACCAGCATGGCACATGTATACATATG
TAACTAACCTGCACAATGTGCACATGTACCCTAAAACTGAGAGTATAATAAAAAAAA
AAAAA

Reverse:
TATCTAGCTTGCCTTTGGCATCTTACCTCGCTAGATGTTAAAAAAAACTATATGTAACA
CAACCTAATTTTTTCTTTTTTTTTTTTTTTTTTTTTTTTTTTTTTTTTTTTTTTTTTTTTTTTT
TTTTTTTTTTTTTTTTTTTTTTTTTTTTTTTTTTTTTTTTTTTTTTTTTTTTTTTTTTTTT

Reverse complement of reverse
AAAAAAAAAAAAAAAAAAAAAAAAAAAAAAAAAAAAAAAAAAAAAAAAAAAAAAAA
AAAAAAAAAAAAAAAAAAAAAAAAAAAAAAAAAAAAAAAAAAAAAAAAAAAAAAAG
AAAAAATTAGGTTGTGTTACATATAGTTTTTTTTAACATCTAGCGAGGTAAGATGCCAA
AGGCAAGCTAGATA

Amplified sequence as predicted by polished sequence

see key for meaning:
Key:
Forward primer
Reverse primer
Sequence from forward sanger sequencing
Sequence from reverse Sanger sequencing
Sequence from both Sanger sequencing

AACAATCACAATGTAGAAGGGGGGATAGCATTGGGAGATATACCTAATGCTAGATGAC
ACATTAGTGGGTGCAGTGCACCAGCATGGCACATGTATACATATAACTAACCTGCACA
ATGTGCACATGTACCTAAAACTGAGAGTATAATAAAAAAAAATAAAAAAAAAAAAA
AAAGAAAAAAAAAATTAGGTTGTGTTTATATAGTTTTTTTTAACATCTAGCGAGTGTA
AGATGCCAAAGGCAAGCTTAGATAAAAACCACTGTAAAACTGATTTCATTATAACACC
AGTGTAGAATCCAGGCA
